# Research Progress of Automated Visual Surface Defect Detection for Industrial Metal Planar Materials

**DOI:** 10.3390/s20185136

**Published:** 2020-09-09

**Authors:** Xiaoxin Fang, Qiwu Luo, Bingxing Zhou, Congcong Li, Lu Tian

**Affiliations:** 1 School of Electrical Engineering and Automation, Hefei University of Technology, Hefei 230009, China; 2018110395@mail.hfut.edu.cn (X.F.); cc.li@mail.hfut.edu.cn (C.L.); 2 School of Automation, Central South University, Changsha 410083, China; 2018110394@mail.hfut.edu.cn; 3 Powder Metallurgy Research Institute, Central South University, Changsha 410083, China; eas_luo@163.com; 4 Hunan RAMON Science and Technology Co., Ltd., Changsha 410100, China

**Keywords:** automated visual inspection, image detection, surface defect detection, metal planar materials

## Abstract

The computer-vision-based surface defect detection of metal planar materials is a research hotspot in the field of metallurgical industry. The high standard of planar surface quality in the metal manufacturing industry requires that the performance of an automated visual inspection system and its algorithms are constantly improved. This paper attempts to present a comprehensive survey on both two-dimensional and three-dimensional surface defect detection technologies based on reviewing over 160 publications for some typical metal planar material products of steel, aluminum, copper plates and strips. According to the algorithm properties as well as the image features, the existing two-dimensional methodologies are categorized into four groups: statistical, spectral, model, and machine learning-based methods. On the basis of three-dimensional data acquisition, the three-dimensional technologies are divided into stereoscopic vision, photometric stereo, laser scanner, and structured light measurement methods. These classical algorithms and emerging methods are introduced, analyzed, and compared in this review. Finally, the remaining challenges and future research trends of visual defect detection are discussed and forecasted at an abstract level.

## 1. Introduction

Metal planar materials (e.g., steel, aluminum, copper plates and strips, etc.) are widely used in automobile manufacturing, bridge construction, aerospace, and other pillar industries, which make immense contributions to the modern social development and the betterment of life. Nevertheless, in the actual industrial production process, the processing equipment damage or the harsh industrial environment will inevitably lead to certain quality problems of metal planar materials products. Some products surface defects showing large-area or periodic characteristics not only impact on the subsequent production but also threaten the quality of terminal products, which bring huge economic and reputational losses to the manufacturing enterprises. The number, degree, and distribution of surface defects areas are significant factors to determine the quality of industrial metal planar materials. The damage detection methods based on vibro-acoustic modulation [1], wireless sensing technology [2] and other different principles have been researched for a long time. However, the computer-vision-based surface defect detection methods are the most commonly used to find and locate the abnormal areas on the image surface due to their advantages of low cost, easy operation, and superior performance, etc. Nowadays, with the rapid development of hardware facilities and the continuous advance of artificial intelligence technology, automated visual inspection (AVI) equipment has gradually become the standard configuration for industrial manufacturers to improve product quality and production efficiency [3,4,5,6,7,8].

The metal planar materials such as steel, aluminum, copper plates and strips along with the similar appearance characteristics have the unified quality requirement that can be summarized as “precise size, homogeneous, and finish surface”, which means that the thickness and width of the metal planar materials should meet the specified precision requirements, and the surface should be clean and free of scales, cracks, scratches, roll marks and bubbles, etc. The defects on the surface of planar materials not only damage the appearance of planar products but may become the weak link of stress concentration as the source of cracking and corrosion. The AVI equipment for surface defects of metal planar materials has two main functions: defect detection and defect classification. The former aims to accurately detect and locate defects on-line in the industrial production process without identifying the types of defects, so that the industrial site can adjust the corresponding continuous casting/rolling equipment according to the degree and frequency of defects, so as to lose no time in controlling the massive diffusion of similar defects and effectively avoid economic losses caused by quality problems. Meanwhile, defect classification is to recognize and label the detected defects to support the grading of finished products. The classification accuracy is directly determined by the precision of defect detection; thus, the overall performance of an AVI system is mainly limited by the accuracy, time efficiency, and robustness of various algorithms in the process of defect detection, which is the focus of this paper.

The online surface defect detection of metal planar materials faces the following severe challenges in the production process:

(1) High surface reflectance

The surface of steel, aluminum, copper strips, and other extremely thin strips is smooth, and their high surface reflectivity is liable to bring high light and shadow area; then, the phenomenon of inconsistent gray value increases the possibility of false edge detection.

(2) Pseudo defect interference

Pseudo defects such as water droplets, water cloth, rain line, water mist, and other real defects produced in the process of laminar cooling lead to frequent false alarms of AVI equipment [9].

(3) Random elastic deformation

Caused by continuous rolling equipment vibration, roll speed differential, side guide plate heterotopia, rolling speed fluctuation, atmospheric turbulence-like effect, and so on, the random elastic deformation presents random image distortion on the charge coupled device (CCD) camera side [10].

(4) Massive image data

The high speed of the real-world production line, the fast pace of coil change, and the demand for fine defects detection in hot rolling mill make the image acquisition front-end continuously generate massive image data, and its peak speed is as high as 5.12 Gbps [9], which requires that the detection algorithm must achieve a good balance among the detection accuracy, calculation, and reliability.

**Figure 1:**
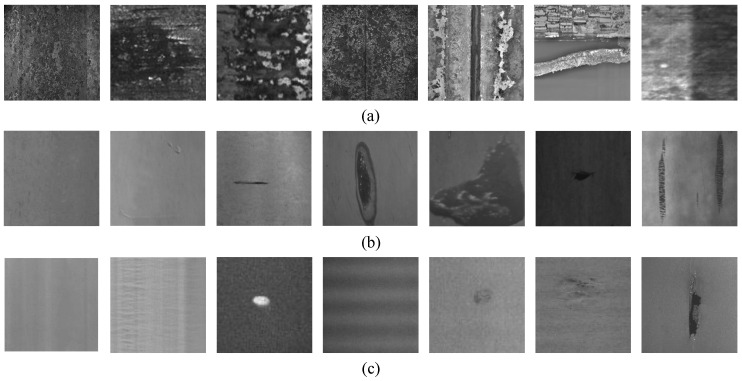
Three classes of typical metal planar materials images: (**a**) continuous casting slabs; (**b**) hot-rolled strips; (**c**) cold-rolled strips.

In different processes, the process operations of different types of industrial equipment make the surface textures have great differences, and the corresponding metal plates and strips also have different detection difficulties. Figure 1 lists three typical surface images of metal planar materials; from the left, the first column includes the defect-free images, and the right side shows several typical defect or pseudo defect images, such as scales, slag marks, cracks, scratches, burrs, and uneven illumination on the continuous casting slab surface; roll marks, scratches, entrapped slag, inclusions, holes and oxide scales on the hot-rolled strip surface; and pickling, corrosions, ripples, stains, pits, and holes on the cold-rolled strip surface. These images are all acquired from the real-world production line using a linear array scanning CCD camera [11].

This paper focuses on the latest theory and algorithm of two-dimensional (2D) defect detection methods based on AVI, as well as the attempt and development process of three-dimensional (3D) technologies in the field of surface defects detection, which will serve as an important reference and inspire scholars to develop methods more suitable for various application scenarios.

The content of this paper is arranged as follows. Firstly, the research background and challenges of the project are introduced. In the second section, the hardware facilities of the 2D surface quality inspection system are described in detail. In the third section, some previous related survey papers are briefly reviewed. The fourth section presents several commonly used performance evaluation criteria for industrial surface defect detection tasks. In the fifth section, four kinds of 2D defect detection methods are introduced in detail, and the classical algorithms and new methods in each category are introduced, analyzed, and compared. Then, in the sixth section, the imaging equipment and detection algorithms of 3D defect detection technology are introduced. Finally, the challenges and future research trends of defect visual inspection are discussed and prospected.

## 2. Two-Dimensional Surface Quality Inspection System

The traditional manual visual inspection methods with the disadvantages of low detection rate, poor real-time performance, low detection confidence, and poor environmental adaptability are not sufficient for the requirements of high precision and fast speed of industrial surface defect detection. With the rapid development of machine vision technology, the automated surface defect detection methods based on machine vision have gradually become the mainstream methods and have been widely used in the surface defect detection of glass bottles [12], mobile phone screens [13], automobile carbon brushes [14], polysilicon solar cells [15], especially steel plates and strips [9], aluminum plates and aluminum strips [16], copper plates and copper strips [17], and other metal planar materials. A machine vision inspection system usually includes an image acquisition subsystem and region of interest detection (ROI) subsystem. Specifically, the camera is used to photograph the product surface, and then the computer system processes and identifies the acquired images to determine whether there are defects on the product surface. This system is highly advantageous for non-contact, high detection accuracy, low cost, and high automation degree.

The hardware of the image acquisition subsystem usually consists of lighting, a lens, and a sensor. Lighting conditions have a great impact on image quality; favorable illumination helps to reduce noise, shadow, and reflection and enhance image contrast, so as to improve the success rate of image processing and analysis, and the commonly used light sources are incandescent lamps, fluorescent lamps, halogen lamps, etc. The lens focuses the light reflected from the surface of the target object to generate a clear image on the sensor. The most momentous part of a camera is the digital sensor, which converts the image into analog or digital video signals. The two main sensors are the CCD (charge coupled device) and CMOS (complementary metal oxide semiconductor). The communication interface receives digital signals and converts them into images in computer memory. An ROI detection subsystem is used to determine whether there are defects on the image surface. It originates in the visual attention technology, which can quickly focus the vision on important targets. In the past 30 years, numerous scholars have carried out in-depth research on ROI detection algorithms, and in the fifth section, we will mainly overview these algorithms.

**Figure 2:**
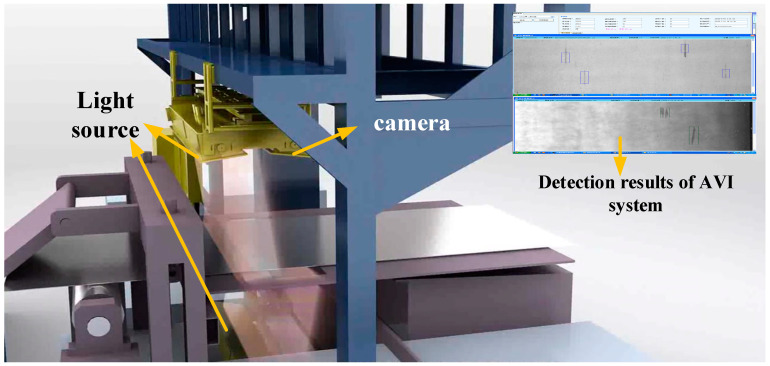
A typical industrial strip surface defect detection system.

Taking the strip surface defect detection system as an example, its hardware framework mainly consists of lighting facilities, a CCD camera, and an image processing computer and server, and its lighting facilities adopt a special infrared light source array. The CCD line scanning camera group is arranged horizontally on the strip steel production line, and the horizontal and vertical visual ranges overlap each other to ensure no missing inspection. The images collected by the CCD camera are transmitted to the image processing computer group via the optical fiber for image processing and pattern recognition. Then, the results, together with the relevant information of the production line are sent to the server database for further processing, and various on-site production information statistical reports are generated. Users can evaluate the quality grade of steel coils according to these reports or analyze the causes of the abnormal production line, so as to realize the real-time monitoring of the production line. Figure 2 shows a typical industrial strip surface defect detection system.

In recent years, great breakthroughs have been made in image processing, computer vision, artificial intelligence, and other related fields, which makes the surface defect detection systems of industrial metal planar materials continuously optimized. In 1900, Matti et al. developed a prototype of the automated online visual inspection system, which collected images by a CCD linear scanning camera, concentrated a strong field light source, and detected defects by combining morphology and statistical algorithms. This system was successfully applied to the surface defects detection and classification of copper alloy strips [18]. In 1995, POSCO along with the Germany Parsytec company developed the automated optical inspection (AOI) system and installed it on the rolling steel strip production line [19]. In 1998, the Parsytec’s HTS-2 system proposed by Rinn et al. firstly employed a high-resolution CCD combined with bright and dark field illumination for surface analysis, which greatly improved the image quality to a large extent [20]. Subsequently, researchers in related fields reported their latest technologies and achievements. Zhang and Ding et al. [21] installed a polarized light filter in front of the camera and rotated the filter in the way of suppressing polarized light to suppress partially polarized light due to metal reflection, thus preventing the image quality from being unsatisfactory. Zhang et al. [16] realized high-speed image processing and data transmission by industrial personal computer (IPC) array, machine vision technology, and Gigabit Ethernet, which can effectively detect defects such as holes, bubbles, foreign matters, and roll marks on cold-rolled aluminum plates. Luo [9] developed a low-cost real-time AOI system based on an embedded image processing board for the automated on-line quality inspection of the hot-rolled strips. As a tool to improve the manufacturing process and industrial quality control, the AVI system has developed well in hardware development, but there is still a broad space for algorithm research.

## 3. Previous Review

Over the past 30 years, researchers have conducted in-depth researches on AVI, and various theories and algorithms have emerged in an endless stream. Some scholars have summarized and compared the relevant research results [22,23,24,25]; however, most of them are relatively old. Recently, researchers have increasingly focused on planar materials, the reviews they presented including those for a particular material (e.g., fabric [26,27] and semiconductors [28]), or those covering a wide range of materials (including fabrics, food, steel, wood, and ceramic tiles) [29,30,31,32]; nevertheless, none of them can specifically review the detection of metal surface defects. In 2014, Neogi et al. [33] made a brief review of steel surface defect detection methods based on AVI, covering the defect detection and classification technology of steel products, including slab, billet, steel plate, hot-rolled strip, cold-rolled strip, and bar. Sun et al. [34] provided a supplement to [33]. However, they all cover a wide range of products and involve defect detection and classification, so it is not highly targeted. It is worth mentioning that Luo and Fang et al. [35] have made a detailed review of two-dimensional visual detection methods for flat steel (including con-casting slabs, hot- and cold-rolled steel strips) surface defects. However, there is still no comprehensive overview that contains both two-dimensional and three-dimension detection algorithms. Related surveys about AVI are listed and compared briefly in Table 1. In order to cover as many plane metal materials as possible with similar surface properties and industrial production requirements and focus on the subject of defect detection, this paper summarizes the surface defect detection methods of steel plates and strips, aluminum plates and strips, and copper plates and strips. On the other hand, the above-mentioned review literature does not involve 3D detection methods for the surface defects of planar materials. In addition to a detailed summary of the 2D detection methods, this paper also introduces the 3D methods, hoping to provide a mite reference for the relevant scholars and engineers engaged in AVI research in the automated manufacturing field.

## 4. Evaluation Criterion

In the task of surface defect detection, we usually evaluate the relevant methods quantitatively according to statistical results, which can be divided into four categories: true positive (TP) indicates the actual defect is detected as a defect, true negative (TN) means the actual defect is mistakenly detected as a background, false positive (FP) means the actual background is wrongly detected as a defect, and false negative (FN) indicates the actual background is detected as a background. Obviously, in the ideal case, the larger the TP and FN, the better the detection effect, while the larger the TN and FP, the worse the detection effect. Nine indicators are consequently defined as follows:

True Positive Rate (TPR):

(1) $$TPR=\frac{TP}{TP+FN}$$

True Negative Rate (TNR):

(2) $$TNR=\frac{TN}{TN+FP}$$

False Positive Rate (FPR):

(3) $$FPR=\frac{FP}{FP+TN}$$

False Negative Rate (FNR):

(4) $$FNR=\frac{FN}{FN+TP}$$

Precision Rate:

(5) $$Precision=\frac{TP}{TP+FP}$$

Recall Rate:

(6) $$Recall=\frac{TP}{TP+TN}$$

Accuracy Rate:

(7) $$Accuracy=\frac{TP+TN}{TP+FP+TN+FN}$$

G-Mean Value:

(8) $$G-mean=\sqrt{TPR\times TNR}$$

F-Measure Value:

(9) $$F-measure=\frac{2\times Precision\times Recall}{Precision+Recall}$$

Among them, G-mean measures the accuracy of these two categories in a combined way, and a larger G-mean means higher TPR and TNR, which is also the requirement of defect detection application. On the other hand, F-measure evaluates the overall performance of defect detection based on the accuracy and recall rate.

## 5. Taxonomy of Two-Dimension Defect Detection Methods

**Figure 3:**
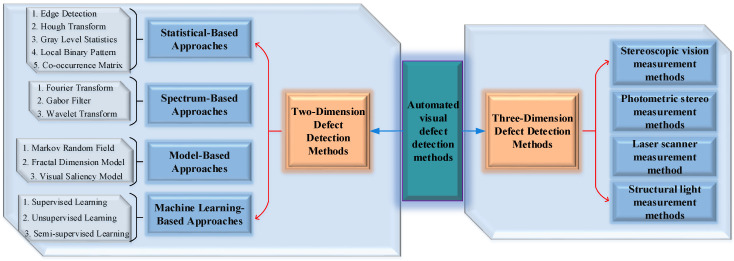
The overall structure of detection method taxonomy.

In this section, the existing two-dimensional vision technologies and models of metal planar materials surface defect detection methods are comprehensively reviewed, and the discussions and prospects are also carried out, the overall structure of detection method taxonomy is shown in Figure 3. Researchers divided the previously proposed methods into different categories according to different characteristics; however, due to scholars’ subjective differences, these categories also vary with each individual. For instance, Zhang et al. [17] thought that texture can be divided into statistical texture and structural texture, and accordingly, the surface defect detection methods based on machine vision are divided into non-texture surface defect detection and texture surface defect detection, in which the former includes threshold methods and pyramid methods, and the latter includes spatial domain methods and frequency domain methods. Yet, based on diverse technology roadmaps, Wang et al. [36] classified defect detection methods into three categories: classification-, local exception- and template matching-based. Youkachen et al. [37] proposed that the detection methods can be classified into a probability model, statistical model, proximity model, deviation model, and network model. Wu et al. [38] divided the methods into statistics-, structure-, spectrum- and subspace-based methods. The texture analysis problem is formerly settled by statistical, spectral, and model-based methods. However, it is noteworthy that the rapid development of deep learning in recent years has changed this pattern; more and more defects detection methods based on deep learning have been applied to metal planar materials. Hence, this paper divides the surface defect detection methods of metal planar materials into four categories: traditional statistical-based methods, spectrum-based methods, model-based methods, and emerging machine learning-based methods. 

### 5.1. Statistical-Based Approaches

From the perspective of statistical methods, the image texture is regarded as a random phenomenon. Statistical methods study the regular and periodic distribution of pixel strength by measuring the statistical characteristics of pixel spatial distribution, so as to detect the defects on the metal planar materials surface. The following is a brief introduction to five representative statistical approaches and the comparison of several typical approaches of these five categories is given in Table 2.

#### 5.1.1. Edge Detection

**Figure 4:**
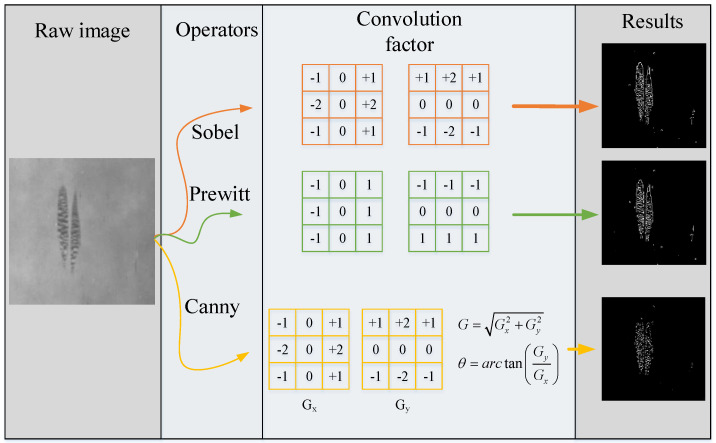
Results of several original edge detection operators.

Edge detection is essentially the detection of grayscale or structural mutation in the tested image. The difference of gray level between the defect area and the background results in an obvious edge at the boundary, which can be applied to detect surface defects of metal planar materials. Owing to the discontinuity of pixels at the edge of the image, researchers usually employ local image differentiation technology to obtain edge detection operators, and the commonly used edge detection templates of metal planar materials surface defects include Prewitt [43], Sobel [39,44], and Canny [45] operators, Figure 4 shows the detection results of these primitive operators on the same defect sample. These operators also have their own shortcomings, and many researchers have optimized them to achieve better results. Table 3 presents the traditional versions and the optimization ones, and it makes a brief comparison of the advantages and disadvantages of these operators. 

In practice, various differential operators are generally used to realize differential operation by a small region template and image convolution, which is extremely sensitive to noise and uneven illumination, so image preprocessing is necessary when using differential operators to detect the edge. Generally speaking, the edge detection operators are deficient in the images with complex gray changes and rich details, and they are even less ideal once the noise interferes. For this reason, some other edge detection methods have been proposed continuously. Zhao and Yan et al. [46] proposed a multi-scale edge detection algorithm based on wavelet transform modulus maxima for the on-line detection of surface defects of a cold-rolled steel strip, which realizes the edge extraction of strip surface defects on multiple scales by balancing edge detection accuracy and noise resistance. Furthermore, edge projection profilometry, a non-contact and non-interference measurement technology, has been widely applied to macro-scale surface profile detection and sub-millimeter scale feature detection [47], and it has broad exploration space in the application of surface defect detection of metal planar materials. The application of these methods in the surface defect detection of metal planar materials is worth exploring. In addition, the fitting operator first uses the edge parameter model to fit the local gray value and then carries out edge detection on the fitting parameter model to smooth noise, which has a certain research value.

#### 5.1.2. Hough Transform

Hough transform (HT) utilizes the global characteristics of the image to detect the target contour directly and connect the edge pixels to form the region closed boundary. As for the application on metal planar materials, Sharifzadeh et al. [40] utilized HT to detect holes, scratches, coil breakage, and corrosion on a cold-rolled steel strip, and Bulnes et al. [48] applied HT to detect the straight line of a strip edge. Given the shape of the region in advance, HT conduces to obtain the boundary curve and connect discontinuous boundary pixels, but the uncertainty of the surface defect shape of most metal planar materials often leads to unsatisfactory detection accuracy. In addition, HT appears to have strong anti-interference ability in line detection, which helps to suppress the influence of nonlinear structures such as noise and incomplete edges, but the complexity of HT increases at a certain rate when encountering massive parameters.

#### 5.1.3. Gray-Level Statistics

Due to the uneven reflection caused by the surface morphology and texture as well as the change of ambient light, the pixel intensity difference between the defect area and background is quite large. The probability distribution of pixel intensity is estimated from a set of images without defects, which is used to select pixels with outliers as candidate defects pixels. Choi et al. [49] first employed the spectrum-based method to obtain the distribution estimation of the image background and then locally refined the defect area to obtain probability estimation, and the detection results were still robust in the noise environment. In the current industrial plate and strip defect detection tasks, the pixels in the detected surface image are regarded as the realization of a random process described by a certain probability distribution. Before defect detection, it is necessary to determine the probability distribution of pixel intensity in the tested image, and Gaussian (normal) distribution and Weibull distribution are two common cumulative distribution functions [50]. Zhao et al. [51] assumed that the background intensity of the aluminum foil image obeys Gaussian distribution, and the distribution of defect parts was different from the background, which was determined by a chi-square test of unknown input estimated value. The processing time of each image is less than 52 ms, and the correct precision rate of 95% is achieved on the production line running at the speed of 5m/s. Wang et al. [36] studied the statistical characteristics and intensity distribution of a large number of defect-free images of strip steel and found that the Gaussian function can fit the histogram of defect-free images well. In addition, the authors also established a unique guiding template, which can accurately locate defects through subtraction operation, reverse sorting operation, and adaptive threshold determination between the guide template and the sorted test images (both accuracy and recall rate are higher than 0.95). Fofi et al. [52] put forward a new, nonparametric, and efficient defect detection method based on Weibull distribution, which calculates two parameters of a Weibull function to fit the distribution of the image gradient in the local area. This unsupervised method has achieved good test results in the industrial optical inspection database containing challenging planar materials defect images, but Weibull is powerless for defects with a gradual pixel intensity or low contrast. Liu et al. [53] proposed a Haar Weibull variance (HWV) model, in which the Haar feature of the local image block replaces the feature of local gradient amplitude. At the same time, the detection accuracy rate and recall rate of this method on the uniform texture defect dataset collected from the actual hot-rolling mill exceed 96%, and the calculation time on each test image of 864 × 864 pixels is 52 ms. Furthermore, in order to increase universality, Ma et al. [41] combined the advantages of global and local features and proposed a neighborhood gray difference method based on multi-directional gray-level fluctuation, which also achieved higher surface defect detection accuracy.

#### 5.1.4. Local Binary Pattern

**Figure 5:**
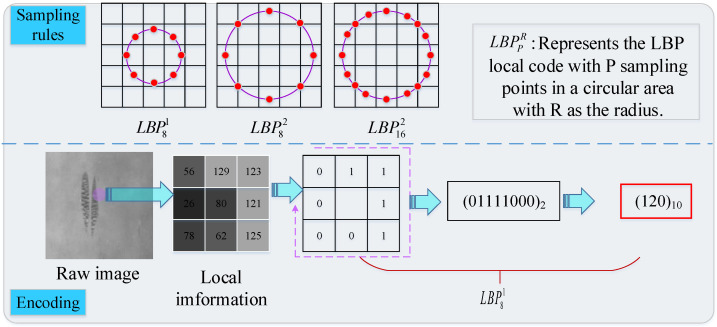
The sampling and encoding rules of traditional local binary pattern (LBP).

Local binary pattern (LBP) marks the difference between the center pixel and its neighborhood pixel by threshold, and it is commonly used in image local feature comparison. LBP proposed by Ojala et al. [54] in 1994 is a simple yet effective texture operator, and the coding and sampling rules are shown in Figure 5. Song et al. [55] summarized texture features based on LBP and their applications in texture analysis, face recognition, and other fields. In order to overcome the shortcomings of the initial LBP, such as weak global description ability and noise sensitivity, researchers have constantly proposed new LBP variants. As shown in Table 4, the comparison of tradition LBP and its variants applied on detecting defects on metal planar materials is given. For example, for the surface defect detection of strip steel, Wang et al. [56] proposed a feature extraction method based on LBP that simultaneously calculates the changes of the horizontal direction, vertical direction, and two diagonal directions, by which the feature extracted has better visual recognition ability, but this method still traps in the problem of noise sensitivity. Based on this, some researchers changed the threshold mechanism for neighborhood evaluation to enhance the anti-noise ability of LBP [6]. However, similar to CLBP, its scale adaptability is not prominent. Chu et al. [57] proposed a smooth local binary pattern (SLBP) and combined it with GLCM, which can not only effectively suppress noise but also extract features with rotation, illumination, and translation invariance. All LBP algorithms focus on one point when they operate on the test image, but different defects have different sizes, and the corresponding appropriate scale to describe the texture features is different, so different block sizes should be selected. Multi-scale local binary mode (MB-LBP) changes the block size to express the defect characteristics with an appropriate scale to ensure the detection accuracy [58,59,60]. It cannot be ignored that the useful description information in the non-uniform patterns in all these LBP variants has been elided. Luo et al. creatively used the reverse thinking to explore the non-uniform pattern to supplement the description information hidden in the uniform pattern in [7] and [61]. As lightweight feature descriptors, LBP and its variants can be applied to defect detection and classification at the same time, and researchers should follow and develop LBP variants or LBP-like descriptors with better noise robustness and scale invariance, which is also in line with the future development trend of AVI.

#### 5.1.5. Co-Occurrence Matrix

Gray level co-occurrence matrix (GLCM) describes the spatial dependence of pixel gray level, which can be used to extract the feature parameters of image texture on the surface of the plane workpiece, and these feature parameters are significant information for analyzing image primitives and arrangement structure. In 1973, Haralick et al. [62] first proposed a GLCM with 14 texture features to reflect the comprehensive information of image grayscale about direction, adjacent interval, and change amplitude, which owns powerful automated texture recognition ability. Nevertheless, balancing matrix performance and window size is a difficult assignment. In order to overcome the limitation of GLCM only describing local features, Wang et al. [42] combined the histogram of oriented gradient (HOG) with GLCM to describe the global and local texture of steel surface images, respectively, but this method is very sensitive to background noise and non-uniform gray changes, and its calculation is complex. Tsai et al. [63] take the weighted eigenvalue of GLCM as a single discriminant feature, thus achieving low computational complexity and strong noise robustness at the same time.

### 5.2. Spectrum-Based Approaches

Many statistical methods are not reliable in the case of illumination variation and pseudo defects interference. Fortunately, researchers have found that images are more likely to be separated out information of different characteristics in the transform domain, and it is possible to find a better defect detection method than the direct processing method in the pixel domain. Some transformation domain methods are summarized as bellow and the comparison of several typical approaches of these categories is given in Table 5.

#### 5.2.1. Fourier Transform

Fourier transform can transform images into the frequency domain and represent texture by spectral energy or spectrum entropy, which realizes translation invariance, unfolding invariance, and rotation invariance. The defect images directly obtained from the steel production line normally contain background noise; therefore, for removing the noise, Yazdchi et al. [67] used the Time Fourier analysis method to eliminate the black and white vertical strips in the image caused by the steel plate reflecting the ambient light. By calculating discrete Fourier transform (DFT), Paulraj et al. [68] converted the vibration signals generated by the pulse signal acting on steel plates into the frequency domain signals, identified the spectral band, extracted the spectral energy as a feature, and linked the extracted features with the health state or defect state of the steel plates. In order to detect the longitudinal cracks on the surface of the continuous casting slab under complex background, Xu et al. calculated the Fourier amplitude spectrum of each subband in reference [64] and obtained the translation-invariant characteristics.

#### 5.2.2. Gabor Filter

Fourier transform plays an important role in the frequency domain analysis of the whole time period, but it lacks the ability to describe the spatial local information of the signal, so most of the local description information is ignored in the spatial domain. The Gabor filter can effectively make up this disadvantage by modulating Gaussian kernel function on a sine wave of a specific frequency in the spatial domain and frequency domain. On the basis of the Gabor filter, Yun et al. [69] detected thin cracks and corner cracks of coarse steel blocks by minimizing the cost function of energy separation in defect and non-defect areas, and the detection accuracy was 91.9% and 93.5%, respectively. Park et al. [70] used the Gabor filter to filter the hot plate images and enhanced the stripe pattern on the images by adjusting the frequency and direction of the Gabor filter according to the stripe mode of the lighting system. Since the layered defects are difficult to identify, Wu [71] took the Gabor filter method to extract Gabor features from the collected layered defect copper strip images, and after improving the image contrast, denoising, and binarization, the defect area can be efficiently detected. The real part and imaginary part of the typical Gabor detector can be used for image smoothing and edge detection respectively, whose parameters are mainly determined by the size and direction of defects, and a single Gabor filter is hard to obtain ideal results for mixed defects of various sizes. Choi et al. [72] proposed a dual Gabor filter combination method based on morphological feature enhancement to identify small holes in the steel plates. Medina et al. [73] fused Gabor features with other classic image features and greatly improved the image defect detection accuracy. In addition, reference [73] also pointed out that real-time performance should be highly valued in the field application of defect detection in industrial manufacturing, which was realized in the reference [74] that proposed a detection acceleration method based on Log-Gabor filter banks. If Gabor parameters are not selected properly, the boundaries between the defects and the background are not very clear, which will make the defect and background unable to be completely separated. In view of this situation, Wu adopted a Gabor filter model [75] that is optimal and practical under uneven illumination conditions for copper surface defect detection. Similarly, to avoid this parameter selection problem, Tolba et al. [76] developed a new image visual quality measurement method based on a multi-scale structure similarity index, which not only has scale invariance but also achieves high detection accuracy and quasi-real-time processing speed. However, due to the non-orthogonality of Gabor, there is redundancy in different feature components, which leads to the low efficiency of texture image analysis.

#### 5.2.3. Wavelet Transform

**Figure 6:**
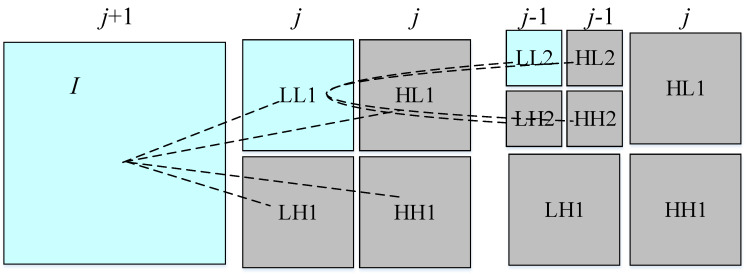
The second-order wavelet decomposition of a two-dimensional image.

Admittedly, the performance of Fourier transform in local feature analysis is unsatisfactory. The Gabor filter overcomes the drawback to a limited extent, whereas the immutability of the sliding window function determines the fixity of the time-frequency resolution. Compared with the Gabor filter, wavelet transform has a more powerful adaptive ability and is in line with human visual characteristics ideally, which could not only locomote the time-frequency window but also automatically modify the window size according to the variation of the window center frequency [77,78]. Figure 6 shows the schematic diagram of the second-order wavelet decomposition of a two-dimensional image, which is decomposed from scale j + 1 to scale J and then to scale J - 1. The result of wavelet decomposition is to divide the image into a collection of sub-images. As a result, to extract information from the signal effectively and analyze the function or signal, scale transform and shift operation have become a prominent advantage of the wavelet transform. In the actual production line, the detection of surface defects of a metal plate and strip is more and more challenging due to the defects such as water drops, oxide scale, uneven illumination, or adverse environment. Jeon et al. [79] proposed an algorithm based on the combination of discrete wavelet transform and morphological analysis to detect corner cracks of an iron oxide scale on the surface of steel billets, which successfully distinguished corner cracks and false defects. Haar, Daubechies2 (DB2), daubechies4 (DB4), biorthogonal spline (Bior), and multi-class wavelets are used to extract the features of small size image blocks by Ghorai et al. [5]. The successful application of the method in the hot strip production line with a rolling speed of 5 m/s completes the detection task at the fastest cost of 16.4 s, which is shorter than the specified 20 s. In order to overcome the lack of resistance to uneven light, Zhang et al. [17] detected copper strip surface defects based on wavelet multivariate statistics. Daubechies wavelet basis is employed to decompose the image scale, and then Hotelling T2 is applied for the statistical analysis of wavelet coefficients. The application of this innovative thinking makes the detection performance of the algorithm better than that of the traditional algorithm. Yan et al. [80] developed a new wavelet image filtering algorithm based on anisotropic diffusion, which extracted defects reliably from the noise background. Wu et al. [66] focused on an undecimated wavelet transform (UDWT) to solve the false alarm problem caused by oxide scale and scale. Moreover, Song et al. [81] adopted the scattering convolution network (SCN) based on wavelet transform to improve the adaptability to local and linearized deformation and successfully applied it to the detection of surface defects of hot-rolled strip steel.

### 5.3. Model-Based Approaches

In addition to statistical- and spectrum-based methods, there is a type of approach with a different work scheme, which is called the model-based approach. The model-based method projects the original texture distribution of the image block to the low-dimensional distribution through the special structure model enhanced by parameter learning, so as to detect various defects better. Several model-based approaches are briefly discussed below and the comparison of several typical approaches of these categories is given in Table 6.

#### 5.3.1. Markov Random Field

In 1983, Cross et al. [84] regarded the texture image as a random two-dimensional image field when they first used the Markov random field (MRF) model. By assuming that the gray level of the pixel is only related to the gray level of the pixel in the field and using the conditional distribution description of the local neighborhood as the local feature of the corresponding random field, the spatial correlation of the image neighborhood pixels is well represented. Furthermore, Gayubo et al. [85] used MRF to repair surface crack defects of metal planar materials and effectively eliminate pseudo features. Starting from the physiological structure of the human visual system, Zhang et al. [86] introduced the observable MRF model into the simulated task-driven attention mechanism and considered the top–down attention and bottom–up attention to complete surface defect detection. Based on the assumption that the correlation of wavelet coefficients of strip surface images of different scales meets the Markov property, Xu et al. [82] proposed an adaptive hidden Markov tree model (CAHMT), which significantly reduced the detection error rate from 18.8% to 3.7%. Recently, studies show that MRF contains great application potential in the detection of industrial surface defects due to its ability to relate local characteristics with global characteristics and good anti-noise performance.

#### 5.3.2. Fractal Dimension Model

Mathematically speaking, fractals are extremely complex and fragmented geometric figures with inherent laws, and fractal dimension (FD) has ideal self-similarity—that is, the overall information can be represented by local features. The grayscale statistics of defect images actually contain features of FD’s self-similarity; Yazdchi et al. [67] applied the multifractal dimension to separate and identify the defect areas of five typical defects on the steel surface. Using the fractal characteristics of digital images, Zhiznyakov et al. [87] detected the defects on the surface of the strips by representing the internal distribution of self-similarity and the image segment with the highest similarity. The experimental results were basically consistent with the detection data of nondestructive testing personnel. Shi and Qiao achieved a new surface fractal dimension, edge circumference dimension (EPD) [88], and established a window dimension trajectory (EPD-WDL) algorithm based on EPD, which is used for plate structure irregularity or damage recognition. The experimental results demonstrated the advantages of the emerging algorithm in defect location and noise anti-interference ability. However, it should be pointed out that FD is mainly applicable to the detection of defects with self-similarity, leading to certain limitations in its industrial application.

#### 5.3.3. Visual Saliency Model

Research exhibits that the human visual system keeps the ability to quickly search and locate objects of interest in the face of natural scenes. By introducing the visual attention mechanism, namely visual saliency, into computer vision tasks, the difficulties of visual information processing tasks are significantly abated [89,90,91,92]. The visual saliency detection model is a process in which computer vision algorithms are used to predict which information in an image or video receives more visual attention. Yu et al. [93] considered the significance of track defects and the regularity of background when detecting rail surface defects; then, they selected pure phase Fourier transform (POFTs) to locate defects. Similarly, Song et al. [94] calculated saliency mapping of grayscale images in order to detect micro-cracks on the surface of steel beams, and they defined central hole-out as a square template for the convolution of filter operator and binarized saliency mapping. Moreover, the pixel retained after filtering corresponds to the position of micro-cracks. In order to be self-adaptive to the shape, size, and scale of the defects, Zhou et al. [83] put forward a surface defect detection model based on double low-rank and sparse decomposition to detect defects as the saliency part of the image, which is not only robust to noise and illumination inhomogeneity but also highly adaptive to the complex and changeable surface defects of a steel plate. After the model was tested on the Northeastern University (NEU) surface defect dataset, the F measure was 0.606, and the calculation time on each image was 0.713 s. Yan et al. [95] developed a probabilistic saliency framework based on a feature enhancement mechanism for realizing robust defect detection on a micro 3D texture surface of industrial products, which designed the absolute strength deviation and local strength aggregation to represent the initial saliency of the pixel level while all pixels are classified as defective or non-defective. To address the issues of intra-class defects having large differences in appearance while inter-class defects contain similar parts, Song et al. have studied many approaches to combine visual salience with other ideas, such as Encoder–Decoder Residual network (EDRNet) [96], multiple constraints and improve texture feature (MCITF) [97], attention mechanism [98], and pyramid feature (PGA-Net) [99], and the experimental results show that they are both effective and outperform the state-of-the-art methods. The advantages of introducing visual saliency are mainly manifested in two aspects: first, limited computing resources are allocated to more important information in images and videos; second, the results of introducing visual saliency are more in line with people’s visual cognition needs.

#### 5.3.4. Other Emerging Models

In addition to the above model-based defect detection methods, new model-based methods have been proposed continuously in recent years. In Table 7, several emerging model-based methods are summarized. The Gaussian mixture model uses several Gaussian models to characterize the features of each pixel in the image, updates the mixed Gaussian model after the new image is acquired, and judges whether it is a defect by matching each pixel of the current image with the updated Gaussian mixture model, which has the advantages of automated image balance and contrast enhancement [100]. Low-rank sparse representation means finding a suitable dictionary for the information commonly expressed in dense form, which simplifies the learning task and reduces the complexity of the model. In recent years, the model based on low-rank sparse representation is also widely used to detect the surface defects of strip steel, and its effect is particularly outstanding. For the sake of making the model-based methods more universal, Wang et al. [56] and Liu et al. [36,101] utilized the guidance information template and proposed methods with preeminent performance. Moreover, Zhou et al. [102] designed a generic method of automated surface defect detection based on a bilinear model, the method realized end-to-end weak monitoring detection of the hot-rolled strips, glass bulb, and other materials by using only small sample data. The model methods are based on the construction model of images and use the statistics of model parameters as texture features. Further, how to optimize the parameters to make the model have better image information description performance will be the main content of model method research.

### 5.4. Machine Learning-Based Approaches

With the rapid development of artificial intelligence technology, widely applied machine learning has shown good results in various fields. Duan et al. [107] and Zu et al. [108] reviewed the application of machine learning algorithms in the field of control and intelligent video analysis. The surface defect detection methods combined with machine learning have been proposed continuously. In this section, we divide machine learning methods into supervised learning, unsupervised learning, and semi-supervised learning according to the learning mode. The summaries and discussions will be given below, and Table 8 lists several typical methods of these three taxonomies.

#### 5.4.1. Supervised Learning

The essence of machine learning is to analyze and learn data (features) and then make accurate decisions or predictions. In 2005, Liu et al. [109] used a double-layer feed-forward neural network to classify the pixel points of test images into defective and non-defective. The basic idea of this task is actually to dichotomize whether there are defects or not, so it can still be classified as defect detection. The convolutional neural network (CNN) is currently the most commonly used deep learning network based on supervised manner [116,117]. Chen et al. [110] performed crack detection based on convolutional neural network (CNN) and Naive Bayesian data fusion schemes, which are called NB-CNN. Considering the diversity of defect shapes, Zhou et al. [111] improved the Fast R-CNN, selected the K-mean algorithm, generated the length–width ratio of the anchor box according to the size of the “ground truth”, and fused the feature matrix with different receiving domains. This method possesses a better capability of microscopic defect detection, and it can still accurately identify the defect type when the light changes, which is easier to be transplanted to the actual industrial application. Subsequently, with the development and improvement of support vector machines (SVM), this generalized linear classifier, which supports data binary classification commendably, is often widely used to distinguish between defective and non-defective regions [118,119]. Ghorai et al. [5] believe that the performance of classifiers in defect detection depends largely on the combination of features and classifiers. For this reason, they permuted and combined different feature sets (Haar, DB2, DB4) and different classifiers (SVM and vector-valued regularized kernel function approximation (VVRKFA)) and observe the defect detection results. The experiments show that the performance of VVRKFA with first-level Haar characteristics ranks first among all feature classifier combinations. Different from the above defect detection methods, He and Xu et al. [112] reversed the general order of ROI extraction and object classification, and they proposed a new object detection framework: classification Priority Network (CPN). The test images were first classified by multiple sets of convolutional neural networks (MG-CNN), and more sparse and reasonable feature groups were output. According to the classification results, CPN regressed defect bounding boxes from feature groups that might contain defects, which were tested on steel plates and strips respectively, and achieved detection rates of 94% and 96%. However, in the real-world industrial production line, to collect and label a large number of image samples is impractical, and the resulting image samples are more unlabeled. In order to achieve satisfactory results with a small number of training samples, data augmentation as well as transfer learning are the key ingredients for training networks. For instance, Yun et al. [120] used the conditional convolutional variational autoencoder (CCVAE) as the data augmentation method, and various defect images are generated by learning the distribution of the given defect data using CCVAE. The experiments showed that in the case of data augmentation using CCVAE, the accuracy could increase from 96.27% to 99.69%, and the F-measure also increased from 96.27% to 99.71%. By applying transfer learning, Neuhauser et al. [121] used the network weights pre-trained on ImageNet as initial weights for the learning procedure; they leveraged transfer learning to accelerate the training process and to enhance the performance of detecting defects on extruded aluminum. The underlying premise of transfer learning is that the feature extraction function of the network can be extended; if the similarity between the source domain and target domain is not enough, the result will not be ideal.

#### 5.4.2. Unsupervised Learning

In the field of machine learning, using unlabeled image samples to detect surface defects of strip steel is called unsupervised learning, which aims to find sample groups with similar information in input data, so it is also called clustering. Based on the similarity between pixels, the clustering method mines the hidden information in texture images and performs clustering based on the features of pixel points. Then, defect detection is realized by the multi-classification method. The clustering method mines the hidden information in the texture image according to the similarity between the image pixels. In detail, clustering is based on the characteristics of pixels, and then defect detection is realized by the multi-classification method. Bulnes et al. [113] analyzed the characteristics of each defect (such as location and shape) and carried out clustering so as to detect the periodically occurring defects with good noise robustness. However, random industrial liquids and other interference factors increase the difficulty of detection. Zhao et al. [122] developed a two-stage marking technology based on superpixels, which firstly aggregated the pixels into superpixels and then aggregated the superpixels into subregions. The boundary of the superpixel is iteratively updated until pixels with similar visual perception properties are aggregated into a superpixel. Then, the author created a method called “L-nearest neighbor” to combine the superpixels and the superpixels whose difference values are within the set threshold into subregions. After several rounds of evolution, the subregions will converge to the defect regions. The accuracy rate of 91% in the cold-rolled strip production line proves the detection capability of this method. 

For a long time, Auto-encoder (AE) has represented strong competitiveness due to its potential to solve the above problems, but the redundant parameters limit the application of the AE method in 2D image data. To break through this limitation, Tao et al. [123] established a cascaded autoencoder (CASAE) framework for connecting the two subtasks of defect detection and classification in complex industrial environments. In this framework design, the detection task binaries the CASAE prediction results through the threshold module, and then the defect area detector extracts and trims the defect area to obtain the accurate defect contour. Furthermore, Youkachen et al. [37] thought CASAE based on supervised learning would spend a long time in the labeling process, so they creatively built a convolutional automated encoder (CAE) based on unsupervised learning to reconstruct defect test images and then used the reconstructed images to highlight shape features through a simple post-processing algorithm. However, due to the inherent nature of the clustering method, such a method is more suitable for defect classification than defect detection, so it is often used as a major tool for classification. The classification performance of unsupervised learning methods is exceptionally dependent on the quality of the input images and the initial parameter design of the classification model. The present instability of unsupervised learning-based classification methods shows some margins of this research stream.

#### 5.4.3. Semi-Supervised Learning

Compared with the above two categories, the semi-supervised learning method chooses a more compromised route by using both limited labeled samples and a large number of unlabeled samples. The generative adversarial network (GAN) [124], consisting of two deep neural networks (i.e., a generator and discriminator), is a typical semi-supervised learning method. The generator continuously generates new images and feeds them to the discriminator. The discriminator, acting as a binary classifier, is used to distinguish the real images from the generated images. GANs are usually applied to generate defective images to expand the limit of defect samples [114,125]. For instance, Di et al. [8] proposed a semi-supervised learning method based on a convolutional auto-encoder (CAE) and semi-supervised GAN to classify surface defects of steel. CAE was trained through unlabeled data and used as a feature extractor, while GAN was introduced for semi-supervised learning to further improve the generalization ability. In order to increase the adaptability of semi-supervised learning and obtain the best semi-supervised learning algorithm, Berthelot et al. [126] unified the currently dominant semi-supervised learning methods and produced a new algorithm, MixMatch, that guesses low-entropy labels for data-augmented unlabeled examples and mixes labeled and unlabeled data using MixUp. MixMatch has obtained state-of-the-art results by a large margin across many datasets and labeled data amounts. Following the MixMatch rules to conduct sophisticated data augmentation, Zhang et al. [115] introduce a new loss function calculation method and propose a new convolutional neural network based on a residual structure to achieve accurate defect detection. Semi-supervised learning methods combine the prior two methods and use labeled samples and a large number of unlabeled samples to train classifiers. The results obtained by a small sample set achieve a similar performance as those obtained by a large training set.

### 5.5. Brief Summary

To separate the abnormal areas from the image background of different complexity is the significant purpose of defect detection. In this section, the algorithms are divided into four detection methods based on statistical, spectrum, model, and machine learning according to their characteristics, and the existing technologies of each method are reviewed and discussed. In addition, the fundamental significance of online defect detection lies in the rapid and accurate detection of abnormal areas on the surface of high-speed moving targets, and the high-performance real-time and anti-interference detection algorithm has always been the target of defect detection. Most of the strip surface defect detection algorithms outlined in this section are tested on exposed offline databases. For example, in the literature [127], Song et al. applied an end-to-end defect detection network (DDN), which combined ResNet and RPN for accurate defect detection and location and tested them on the database NEU defect detection dataset (NEU-DET). The experiment indicates that the mean average precision of DDN for defect detection task reaches 82.3. Liu et al. [53] put forward a Haar–Weibull-Variance (HWV) model detection method; they conducted the experiment on the database containing 100,000 defect images and reached the accuracy rate of 96.2% and the calculation time of 52 ms on each 864 × 864 test image, which proves the high calculation efficiency of this method. In particular, the surface defect detection based on the offline databases is much less difficult in terms of stability, robustness, and real-time performance than the surface defect detection based on the actual production line; consequently, the actual application performance of the algorithm cannot be truly and comprehensively demonstrated. 

It is noteworthy that in recent years, the surface defect detection literature of plate and strip steel shows that the research trend has gradually moved from purely theoretical research to field application. For example, Ghorai et al. [5] developed a set of automated visual detection systems for real-time surface image acquisition to detect surface defects of hot-rolled strip steel. The system is capable of surface defect detection for hot-rolled strip with a running speed of 5 m/s, and the testing time of 200 images is only 16.4 s, which is shorter than the industry regulation of 20 seconds. Luo et al. [9] also developed an embedded algorithm hardware-accelerated automated optical detection system for the surface defect detection task of hot-rolled strip steel and tested it on 18,071 continuous images. The accuracy rate of 92.11% and the processing time of each image is 1.180 s proved that the system meets the requirement of the maximum rolling speed of 20 m/s in the industrial field. The harsh industrial environment (such as high temperature, shaking, dust, water drops, etc.) brings a great challenge to defect detection in the actual industrial production line. The high-speed rolling process requires not only high accuracy but also calculation time. To better illustrate the characteristics of each taxonomy, Table 2, Table 3, Table 4, Table 5, Table 6, Table 7 and Table 8 list typical examples of each type of taxonomy and compare their advantages and disadvantages.

The surface defect detection research based on the existing offline database tests the performance of the new proposed algorithm easily and accelerate the iterative updating speed of the theoretical algorithm, and at the same time, rich and diversified data are needed to ensure the overall performance of the algorithm. Unfortunately, the number and variety of samples contained in the steel surface defect dataset are far from sufficient, and there are few publicly available steels surface defect texture databases. Therefore, because there is no uniform database standard, the methods of many articles cannot be compared fairly. The widely used steel surface defect databases include NEU [81], Dragon [11], and RSDDs [128].

(1) NEU database

**Figure 7:**
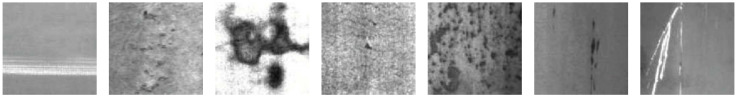
Examples of NEU database.

The NEU database is a public database of surface defects of the hot-rolled strips collected by the Northeastern University research team. Six typical surface defects of the hot-rolled strips are collected, namely, roll-in scale (RS), patch (Pa), crack (Cr), pitting surface (PS), inclusion (In), and scratch (Sc). The database includes 1800 gray images (300 samples for each of 6 typical surface defects). Figure 7 is an example of some drawings of the NEU database.

(2) Dragon database

**Figure 8:**
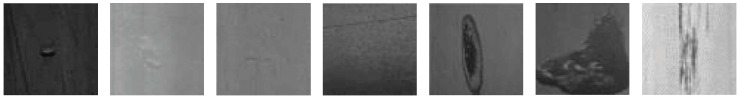
Examples of Dragon database.

Dragon contains 18 categories of defects obtained from an actual hot rolling production line, and each class contains 300 non-overlapping samples. Figure 8 shows a partial pattern example of the Dragon database.

(3) RSDDs database

**Figure 9:**
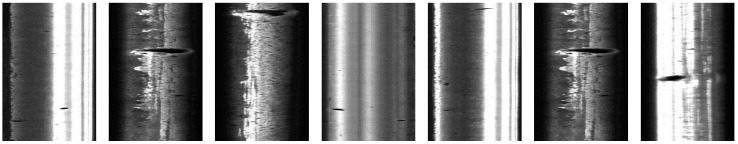
Examples of RSDDs database.

RSDDs consists of two types of datasets: the first type is the type I RSDDs dataset captured from the fast track, which contains 67 challenging images. The second is the TYPE II RSDDs dataset, which is captured from common heavy-duty tracks and contains 128 challenging images. Each image from both datasets contains at least one defect and includes a complex background and various noise. Figure 9 shows a partial sample of the RSDDs database.

## 6. Taxonomy of Three-Dimension Defect Detection Methods

Two-dimensional detection methods mainly rely on gray images to obtain the surface morphology of flat metal and employ gray field change to realize surface defect detection. Due to the lack of height/depth information, these kinds of methods are frequently susceptible to natural light, shadow, water, and oil stains, resulting in false defects. In spite of this, the surface defects of metal planar materials are generally accompanied by height anomalies, such as pits, bumps, depressions, and so on. High reflective spots and dark areas appear in the two-dimensional image after illumination, which is unconducive to the accurate identification of defects. Therefore, the defect detection methods led by 3D imaging technology or 3D reconstruction technology attract wide attention. Making full use of the gray, elevation, and geometric characteristics of the defects, new detection techniques are the development trend of the surface defect detection technology of metal planar materials. 

Three-dimensional (3D) data measurement is commonly divided into contact measurement and non-contact measurement. The former shows the characteristics of high destructiveness, high cost, and slow detection speed, and the latter mainly includes penetration measurement and reflection measurement. Penetrating measurement of radioactive substances causes potential hazards. Consequently, the non-contact and high-security advantages of reflection-type measurement turn into the choice of most people, and outstanding results have been obtained in the surface detection of 3D objects. Non-optical measurement is a type of reflection measurement, from early radar and sonar to ultrasonic imaging [129,130], magnetic imaging [131,132], pulsed eddy current imaging [133,134,135,136], and so on. Non-optical measurement needs to be close to the detected surface and generates blind spots if the rough surface or noise interference occurs. Another momentous reflective measurement is an optical measurement, which is also a 3D technique that will be reviewed in detail in this paper. The number of literatures on 3D detection methods of metal planar materials surface defects is limited, and researchers have different opinions on the classification of optical 3D measurement methods. Pernkopf and O’Leary [137] summed up two range imaging methods: light sectioning and photometric stereo. The former uses projected light to calculate distance, while the latter obtains static scene distance from several grayscale intensity images. Koch et al. [138] divided existing 3D detection research into (1) 3D reconstruction methods using 3D laser scanning and stereo vision and (2) target reconstruction methods based on vibration using acceleration sensors. Song et al. [139] believed 3D information acquisition could be divided into two types: passive stereo vision and active structured light. Passive stereo vision is well applied to areas with large texture variation. Active structured light replaces a camera with a projector and actively projects the required texture on the object surface for stereo matching, which has high spatial resolution and accuracy. According to the method of 3D data measurement, 3D detection technology is divided into four types in this paper: stereoscopic vision measurement, photometric stereo, laser scanner measurement, and structural light measurement, the comparison of these four measurement methods is given in Table 9.

### 6.1. Stereoscopic Vision Measurement Methods

**Figure 10:**
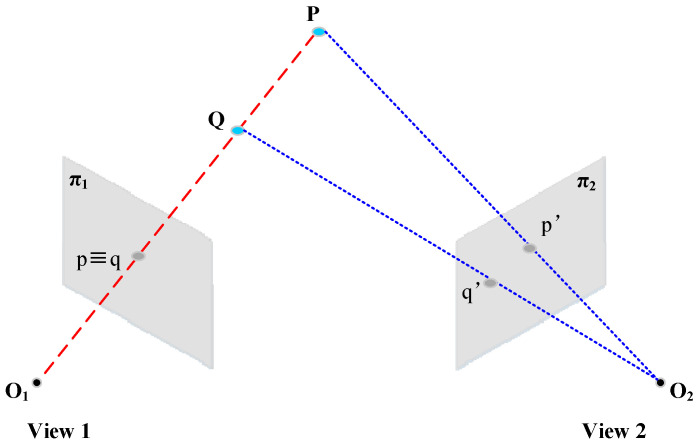
Schematic diagram of stereoscopic vision. P and Q are two objects with different heights; they are imaged at the same point on plane **π_1_** and at different two points on plane **π_2_**.

Non-contact, non-destructive measurement, high precision, high speed, and high reliability are major advantages of the two-dimensional vision system, as an advanced stage of a 2D vision system, passive stereo vision system uses multi-view 2D images for 3D reconstruction; that is, it calculates the relationship between the camera image coordinate system and world coordinate system, and then uses the information in multiple 2D images to reconstruct 3D information. As shown in Figure 10, with two cameras (or more) we can infer depth, by means of triangulation, if we are able to find corresponding (homologous) points in the two images. Monocular, binocular, and multilocular 3D vision measurement systems are developed to meet different application requirements. Wen and Song et al. [140] used binocular stereo vision camera to obtain original gray images and depth images of a steel surface with complex background, false ROI interference, and weak contrast edge, and they established a detection method that fuses gray images and 3D depth information. The superpixel segmentation method maintains the target boundary and accuracy, the compact measurement method of fusion depth information suppresses the clutter background, and the ROI seed extraction and local contrast operation are utilized to obtain a fine significant ROI image. The experimental results show that the running time of this method for a test image is shorter than that of other methods that fuse the 2D image and 3D depth information. Using robots to shoot from multiple perspectives and template matching technology to detect local defects of 3D objects [141,142] is also a general method. For example, Tsai et al. [141] first acquired images of each perspective of a defect-free object, stored them as comparison templates, compared each acquired image with the corresponding template image, and then used template matching technology to identify the local defects of two comparison images in each perspective. For quickly calculating the large dataset of a 3D point cloud, Enzberg et al. [143] provided a reference surface to measure the measured surface with a model-based surface quality detection method, compared its 3D coordinate data with reference data, and accelerated the calculation of 3D point cloud data through dual-eigenvalue decomposition. Niu and Song et al. [144] proposed an unsupervised stereoscopic saliency detection method based on a binocular line-scanning system to detect rail surface defects in complex background information. The experimental results on dataset (RSDDs-113) presented that precision is 0.95, recall is 0.81, and F-measure is 87.16. Stereoscopic vision measurement methods can be well applied to areas with large texture changes and are highly sensitive to normal surface disturbances.

### 6.2. Photometric Stereo Measurement Methods

**Figure 11:**
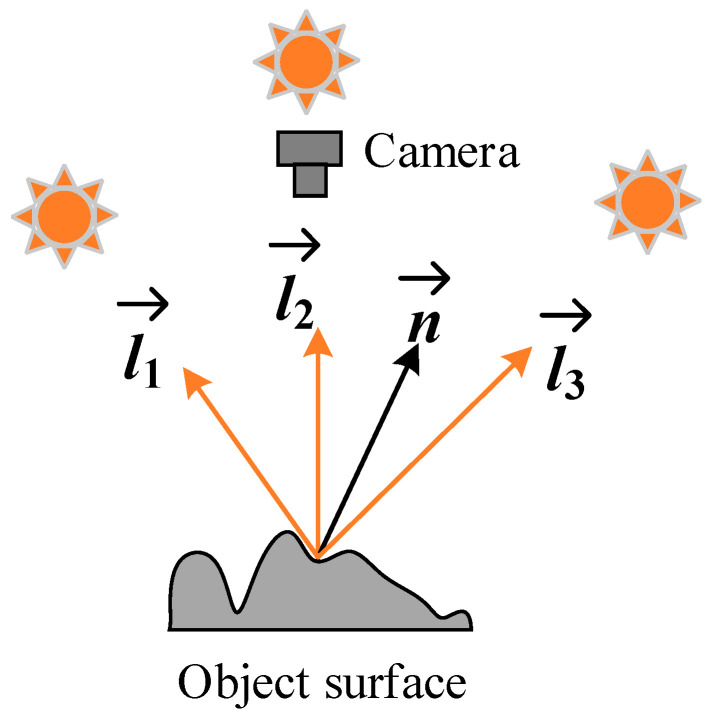
A schematic diagram of the classic photometric stereo measurement with three light sources and a camera.

Photometric stereo refers to a method of estimating the surface normal by using multi-intensity images obtained with different lighting conditions. Figure 11 shows a schematic diagram of the classic photometric stereo principle. The key components include three light sources and a camera. The object to be measured is placed in the field of view of the light source and the camera. The three light sources illuminate the object in turn, and the camera collects three images respectively. Ren et al. [145] used a single camera to carry out three-dimensional reconstruction of multiple images under multiple illumination directions to detect the defects on the smooth steel surface. The camera captures the image of the target surface under different directions, and the surface normals of each point on the target surface are restored by using the pixel values under different light directions, which detects a defect with a diameter of 0.87 mm on the smooth steel surface, which is equivalent to 9 pixels.

Kang et al. [146] developed a plane steel surface detection system based on multispectral photometric stereo vision technology. The surface data were captured by a multispectral camera and an illumination system with different color channels. The surface was reconstructed by integrating the estimated gradient field so that the 3D shape information of the surface could be obtained, and defects with a depth of 0.025–0.48 mm could be detected. The system is first implemented in a three-channel structure and then expanded to a six-channel system, where more than three light sources are used to increase the flexibility of practical applications; for example, highlighting or shading can be ignored when measuring. There is no need for photometric stereo measurement methods to know the precise 3D relationship between the test object and the camera or to use two cameras to capture 3D data.

### 6.3. Laser Scanner Measurement Method

**Figure 12:**
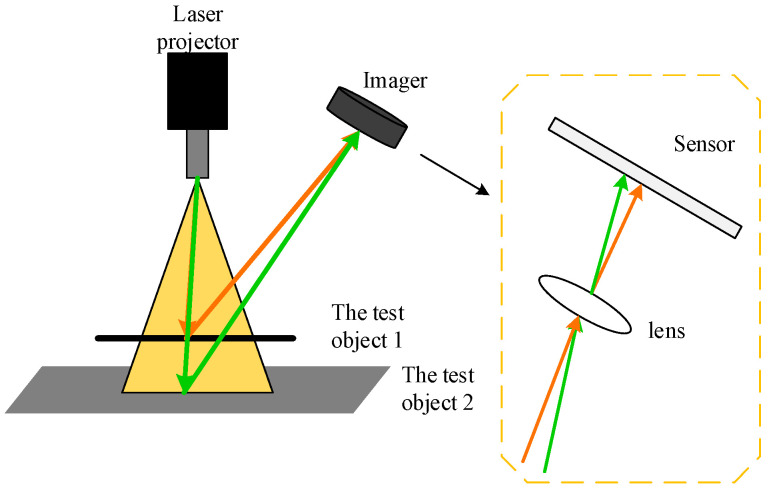
Working principle of triangulation.

Based on triangulation, the laser scanner works by projecting laser points (lines) onto an object and capturing its reflection with a sensor (camera). Millions of 3D point cloud data could be accurately captured to represent the surface of the target object. The working principle of triangulation is shown in Figure 12. Erkal and Hajjar [147] use a camera-integrated laser scanner to capture building surface point clouds, extract local surface features and color information, and more accurately detect cracks, corrosion, and other related defects. Reyno et al. [148] applied 3D laser scanning technology to obtain the geometric point cloud data corresponding to the surface of the object being scanned. The laser point, camera, and laser transmitter formed a triangle, so as to carry out the damage assessment of the aircraft panel. The laser scanner is one of the more common measurement tools in the application field of 3D surface detection of metal planar materials [149,150,151,152,153]. Landstorm and Thurley [150] developed a crack detection strategy for steel plates with 3D surface contour data collected by laser triangulation. The system first uses mathematical morphology to segment the data and then assigns a crack probability to the structural connectivity area by the logistic regression model, which detects 70% of the total length of the manually labeled cracks in the connectivity area. Zhao et al. [151] established a 3D quantitative detection method for surface defects of continuous casting slab using an optimized laser fringe imaging algorithm by using a laser beam and two-beam array charge-coupled cameras (CCD). By comparing CCD camera and image data fusion technology, the optimal imaging method of laser fringe projection on the hot plates surface was studied, and the detection accuracy of 3D shape defects was improved. In order to overcome the weakness that a laser scanner is vulnerable to changes in the surface properties of the object under test, Martin et al. [152] built an intelligent visual detection system based on laser diode diffuse lighting to detect the iron scale defects on the surface of stainless steel. The vision system illuminates the entire detection area with diffuse illumination, shining a high-intensity beam on the surface. The diffuse laser illumination eliminates the shadow generated by the surface roughness in the acquired image, solves the problem of high reflectivity on the surface, and is able to find defects with a diameter of 50 microns on the surface of the stainless steel moving rapidly at 1 m/s. The millions of data points captured by 3D laser scanners are called high-density data. Generalized likelihood ratio (GLR) technology to automatically identify the potential failure of a high-density dataset is most likely related to the position, size, and shape; Wells et al. [153] optimized the generalized likelihood ratio (AGLR) technology to identify the high-density data that are most likely to contain the fault data, which converts the current special visual inspection method to one that is statistically viable to automatically check the solution. The method can almost completely reflect the external geometry of the tested object, and the false alarm rate is only 4.4%. Laser scanners reproduce surface shapes without contact, damage, and accuracy, but there are also challenges such as high equipment cost, large computation, and difficulty in balancing resolution and acquisition speed.

### 6.4. Structural Light Measurement Methods

**Figure 13:**
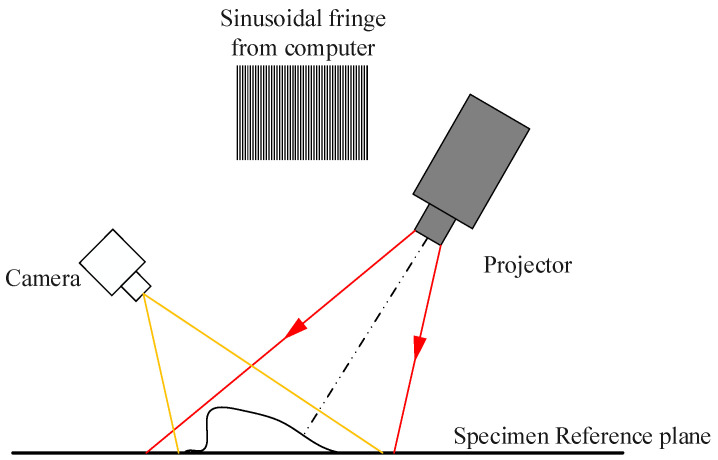
Working principle of a typical surface structure light (grating projection technology).

Different from the single or multiple cameras and laser scanners of the stereo vision method, the active structured light method uses an optical projector to project a certain pattern of structured light onto the surface of an object and then forms a three-dimensional image modulated by the surface shape of the object to be measured. The main principle is shown in Figure 13; a sinusoidal fringe is generated by computer, the sinusoidal fringe is projected to the object to be measured through a projection device, the camera is used to capture the degree of bending of the fringe modulated by the object, and the phase is obtained by demodulating the curved fringe, which is followed by converting the phase into the height of the whole field. Structural optical scanning systems have been applied to many aspects of surface defect detection. For example, Zhang et al. [154] identified the stem and calyx based on the change of image pattern of infrared linear array structural light when it was projected on an apple. Chu et al. [155] used laser projectors to generate a kind of structural light, which was projected onto the welded surface. By calibrating the spatial relationship between the laser projector and the camera, the 3D weld surface was reconstructed. Structured light can be divided into many types; its essence is to structure light. The simple structure includes point structure light, line structure light [156,157], and simple surface structure light; the more complicated structure rises to the coding of the optical pattern. Cao et al. [157] collected the point cloud pattern by high-precession structured laser sensors based on line-structured light, and it can achieve megapixel resolution to meet the precision requirement for rail surface defect detection. Grating projection technology is a kind of widely used structured light that actually belongs to the generalized surface structured light. Grating projection technology is actually a kind of surface structured light in a broad sense, which generates sinusoidal fringe through computer programming, projects the sinusoidal fringe to the measured object through the projection device, uses a CCD camera to shoot the bending degree of the fringe adjusted by the object, demodulates the bending fringe to get the phase, and then converts the phase to the height of the whole field [158,159]. Although the active structured light approach contains many advantages (for example, high spatial resolution and accuracy), it embodies several problems: Firstly, active structured light usually requires spatial or temporal phase unwrapping, which increases the computational complexity and reduces the detection speed. Secondly, the projector is accurately calibrated by an active structured light method. There are many different kinds of projector calibration methods, but accurate projector calibration is still very difficult. In addition, active structured light methods usually need to calibrate the nonlinear projection and compensate for the errors associated with it.

### 6.5. Brief Summary

The stereo vision methods rely on the intrinsic texture information of the object surface; photometric stereo methods have the limitations of detection of non-Lambertian surfaces; laser scanning equipment shows the high cost and a large amount of computation; they are unsuitable for on-line or real-time monitoring of metal planar materials. The projector calibration of the structured light method is complicated and easy to cause errors. In order to solve the above problems, Wen and Song tried to combine the stereo vision method and structural light method to detect the surface height information of high-temperature steel products. They designed a set of static object 3D detection systems [160] and established the mapping from the 3D point of the detection surface to the camera coordinate system through camera calibration and a stereo vision principle by using structural light technology, and they obtained the surface sample height information by using stereo correction and cylinder coding methods. The actual experimental results of this method are very close to the real height, with a minimum error of 1 mm and an average error of less than 2 mm. In addition, the hexagon grid census (Hg census) transform is proposed to improve the robustness of stereo matching. In order to avoid phase unrolling, a passive stereoscopic parallax map was used as the constraint condition to achieve phase matching. Meanwhile, the local phase matching and subpixel parallax thinning method were proposed to obtain higher measurement accuracy [139]. Due to the special surface information acquisition mechanism of the 3D detection method, compared with the 2D detection method, height information is obtained, so defects that cannot be detected by the 2D detection method can be detected and the detection accuracy can be improved. However, 3D information measurement equipment is highly complex, and the magnitude of 3D point cloud data collected is extremely high. Therefore, it is urgent to develop 3D computational imaging technology with high-speed data concurrency and 3D visual detection algorithm with strong timeliness.

## 7. Summary and Discussion

From simple binary image processing to high-resolution multi-gray image processing, and from general 2D information processing to 3D vision processing, machine vision, as an emerging and rapidly developing discipline, has moved from laboratory research to practical application. Different from previous reviews, this paper focuses on planar metal materials with similar quality control requirements, and it is the first one to comprehensively summarize defect detection methods from two-dimensional and three-dimensional aspects. This paper summarizes the research results of the automated visual defect detection of metal plates and strip surfaces in the last 30 years, most of which were published in last 10 years. In Section 5 and Section 6, four kinds of 2D detection technologies and four kinds of 3D detection methods are reviewed respectively. The learning methods, theoretical discussion, and application development of surface defect detection of industrial metal planar materials is introduced and summarized. Based on the inspiration from the above literature and the accumulation of the author’s experience in developing the surface defect detection system [9], the challenges and research suggestions that still exist in this field are listed as follows.

(1) Compared with other surface defect detection tasks based on computer vision, in real-world metal planar materials industrial manufacturing, in addition to the balance between detection accuracy and computational efficiency, the more important thing is to ensure the stability of the detection algorithm, especially the robustness to environmental changes. In addition, it is also necessary to have the ability to detect the diversity of defects, especially deformation defects without edge features. 

(2) The rapidity and generality of the algorithm are two key problems in the application of a real-world AVI system. As for the algorithm itself, compared with a complex learning network, online surface defect detection, as an unsupervised real-time detection task, is more inclined to use a lightweight algorithm, while machine learning or a deep network is more suitable for dealing with complex multi-class classification problems with rich datasets (namely, defect classification). As far as hardware is concerned, the concept of edge computing can be used for terminal acceleration; for example, application specific integrated circuit (ASIC), similar to field programmable gate array (FPGA), can be placed in the front end of image acquisition to complete the preprocessing of original data in real time, so as to prevent the complicated information from affecting the subsequent processing.

(3) Noise smoothing and edge enhancement are important preprocessing operations for defect detection, which should be arranged as close as possible to the sensor side of imaging. In addition, the most effective denoising method of the AVI system is to make the image as clean as possible through some feasible engineering measures. For example, a blower or an ion air gun should be installed on the lens side and the surface side of the target to remove water drops, dust, fibers, etc. on the optical surface caused by the harsh industrial environment, and a light source with uniform and moderate brightness should be applied on the target side to overcome the influence of uneven light caused by the variation of illumination during the day and night. In addition, some cost-effective measures of cooling configuration and security protection for imaging devices are also of great significance for avoiding imaging degeneration caused by harsh environments such as high-temperature and mechanical vibration.

(4) The latest machine learning technology provides a new way to handle this problem of data imbalance. For example, the GAN has achieved great success in generating defect samples. To avoid the problem of poor interpretability in a GAN, it can be connected with reinforcement learning, and a GAN can be applied to reverse reinforcement learning and simulation learning to improve the efficiency of the reinforcement learning, the ability to transform images and texts, and the ability of machines to understand. Since researchers conduct experiments with different methods on different datasets, it is difficult to fairly compare the detection performance of different technologies. Inspired by the research in the field of biometric identification, it will be a long-term work in this field to construct a rich and diversified database of surface defects of metal planar materials. 

(5) The automated visual detection of metal planar materials surface defects should strive to adapt to the new adjustment of world industrial competition pattern and seize the commanding heights of future industrial competition. Efforts should be made to promote the deep integration and organic collaboration of multiple technologies, and the study of metal planar materials surface defect detection algorithms and instruments with high detection accuracy, strong real-time performance, and high stability play an important role in the automated control and whole process management of surface quality of industrial metal planar materials.

## Figures and Tables

**Table 1:** Related surveys about automated visual inspection. AVI: automated visual inspection.

Reference	Years	The Main Contents	Inadequacies
[22]	1982	This paper discussed the problem of automated visual inspection in industry from the aspects of hardware, software, system throughput, universality, and reliability.	These papers only discussed the general advantages and the feasibility of the AVI method, and have been published for a long time
[23]	1988	This paper summarized the progress made by the AVI industry from 1981 to 1987 and the problems to be solved.	
[24]	1995	In this paper, the most suitable algorithms for real-time application were mainly introduced.	
[25]	1995	The general advantages and feasibility of AVI were discussed in conjunction with the literature from 1988 to 1993.	
[26]	2008	The detection techniques based on computer vision were reviewed from the point of view of fabric surface defects.	There is no special review on metal planar materials surface defect detection technology.
[28]	2015	Optical detection systems in the semiconductor industry were reviewed.	
[29]	2008	The research progress of surface detection technology based on texture analysis method in recent years was reviewed.	
[30]	2014	The applications of several typical surface defect detection techniques on multiple surfaces were compared.	
[31]	2017	The applications of machine vision surface defect detection in many kinds of planar materials were reviewed.	
[32]	2020	This paper reviewed the vision-based automated detection methods for metals, ceramics, textiles, and other materials, and it describes the types of defects in detail.	
[33]	2014	This paper summarized the detection methods of steel surface defects based on AVI, including the detection algorithms and classification algorithms of six types of steel products such as slab, strip and bar, and it summarizes the hardware composition of AVI system.	It covers a wide range of products, involving defect detection and classification, which is not well targeted.
[34]	2018	This paper provided a supplement to [33]; it also covers AVI methods of flat steel products and long steel products.	
[35]	2020	This paper made a detail review of two-dimensional visual detection methods for flat steel (including con-casting slabs, hot- and cold-rolled steel strips) surface defects.	Only two dimensional detection methods are involved.

**Table 2:** The comparison of several typical statistical-based surface detection approaches.

Methods	Reference	Approaches	Defect Types	Difficulties	Advantages	Disadvantages
Edge detection	[39]	Eight directional Sobel operator	Backfin defect	Random noise interference	Robust to noise and protect edge shape well	Only suitable for low-resolution images
Hough transform	[40]	Traditional Hough transform	Holes, scratches	Complex background and noise interference	Strong anti-interference ability	Only detects defects of certain shapes
Gray level statistics	[41]	Multi-directional gray fluctuation	Multi-type defects	Complex texture characteristics	Suitable for low-resolution images	Poor timeliness and cannot automatically select the threshold
Local binary pattern	[6]	Adjacent evaluation completed local binary patterns	Multi-type defects	Uneven illumination	Robust to noise	Weak robustness to scale variation
Co-occurrence matrix	[42]	Combination of GLCM and HOG	Scales	Complex texture characteristics	Extracted the spatial correlation between image pixels completely	Computing and storage requirements are relatively high

**Table 3:** Comparison of several edge detection operators.

Reference	Operators	Application	Advantages	Inadequacies
[43]	Prewitt combined with the Gaussian smoothing operator	Aluminum strip	Achieves high robustness to image non-uniformity	The results are not ideal for images with mixed complex noises
[44]	Traditional Sobel operators	Steel sheet	Good detection result for images with gradual gray variation and low noise	Has only two templates for horizontal and vertical edges detection respectively, which has limitations
[39]	Eight directional Sobel operator	Rail	Suppresses false edge detection that is easy to trigger well	The computational burden is relatively high
[45]	Double-threshold Canny operator	Copper strip	Avoid false detection as far as possible	Poor adaptive ability makes it easy to blur the noiseless region sometimes

**Table 4:** Comparison of tradition LBP and its variants applied on detecting defects on metal planar materials.

Reference	Methods	Improvements	Advantages	Disadvantages
[54]	Traditional LBP	-	Rotation and gray invariance	Sensitive to scale variation and noise interference
[56]	Improved LBP	Simultaneously calculate the changes in multiple directions	Has better visual recognition ability	The noise suppression ability is not outstanding
[6]	Adjacent evaluation completed LBP (AECLBPs)	Changed the threshold mechanism of CLBP by taking neighborhood pixels instead of central pixels	Has high recognition accuracy and strong anti-noise ability	Scale adaptability is not prominent
[60]	New multi-scale LBP (new MB-LBP)	Changed the block size and replaced the simple average with the percentage difference between the neighborhood block and the center block	Enhances the robustness to scale variation.	The noise suppression ability is not outstanding
[7]	Generalized complete LBP (GCLBP)	Explore the non-uniform pattern hidden in the uniform pattern	With strong anti-interference ability and simple calculation	It cannot suppress noise and adapt to scale variation well at the same time

**Table 5:** The comparison of several typical spectrum-based surface detection approaches.

Methods	Reference	Approaches	Defect types	Difficulties	Advantages	Disadvantages
Fourier transform (FT)	[64]	Combination of FT and curvelet transform	Longitudinal cracks	Complex background information	Invariant to translation, expansion, and rotation	Background and defect information in frequency domain can easily be mixed to cause interference
Gabor filter	[65]	Traditional Gabor filter	Periodic defect	Uneven illumination	Suitable for high-dimensional feature space	Difficult to determine the optimal filtering parameters and no rotation invariance
Wavelet transform	[66]	Undecimated wavelet transform	Horizontal scratch	Pseudo-noise interference and uneven illumination	Suitable for multi-scale image analysis and can compress image effectively	Difficult to select a proper wavelet base

**Table 6:** The comparison of several typical model-based surface detection approaches.

Methods	Reference	Approaches	Defect types	Difficulties	Advantages	Disadvantages
Markov random field	[82]	Hidden Markov tree model	Multi-type defects	Complex texture characteristics	Can reflect the underlying structure of the image	Not suitable for global texture analysis and small size defects
Fractal dimension model	[67]	Multifractal decomposition	Multi-type defects	Irregular defect shape	Global information can be represented by local features	Only applicable to images with adaptability
Visual saliency model	[83]	Double low-rank and sparse decomposition	Multi-type defects	Mixed pattern information and pseudo-noise interference	Robust to noise and uneven illumination	Limitations on gradient strength or low contrast defects

**Table 7:** Comparison of several emerging model-based methods. FNR: false negative rate, FPR: false positive rate, TNR: true negative rate, TPR: true positive rate.

Reference	Models	Main Content	Performance
[103]	Gaussian mixture model	The Gaussian mixture model and local and nonlocal linear discriminant analysis are combined to solve the problem of dimension reduction and defects detection and recognition.	TPR = 0.993
[104]	Gaussian mixture entropy model	Authors used the non-extensive entropy with Gaussian gain as the regularity index and utilized this entropy for localizing texture defects through Gaussian mixture entropy modeling.	FNR = 0.078
[105]	Smooth and sparse decomposition model	The method exploits regularized high-dimensional regression to decompose an image and separate anomalous regions by solving a large-scale optimization problem.	FPR = 0.010 FNR = 0.004 Time (sec) = 0.195
[106]	Low-rank sparse reconstruction model	The method detects the defect via low-rank decomposition with the help of the texture prior, which is estimated by constructing a texture prior map on the given images where higher values indicate a higher probability of abnormality.	TPR = 0.72 FPR = 0.31 Accuracy = 0.99 Precision = 0.69 F-measure = 0.68 Time (sec) = 0.81
[56]	A concise and compact guidance information model	The authors provided a paradigm of incorporating intrinsic priors of defect images, which detects the surface defects at the entity level rather than pixel level.	FPR = 0.01 FNR = 0.02 Time (sec) = 0.945
[36]	A guide template model	A guide template is proposed to sort the gray value of each column pixel of the test image and use the guide template to subtract the sorted test image to locate defects.	Precision = 0.95 Recall = 0.97 F-measure = 0.96 Time (sec) = 0.035
[101]	A new self-reference template-guided model	The authors calculated the statistical characteristics of a large number of defect-free images and built a specific template for each test defect image. Then, it was based on the self-reference template to detect defects.	Precision = 0.99 Recall = 0.98 F-measure = 0.98
[102]	Bilinear model	The authors designed the dual-vision geometric group 16 (D-VGG16) as the feature function of the bilinear model, used the gradient weighted function class activation mapping to obtain the heat map of the original image, and used the threshold segmentation method to process the heat map and automatically locate the defects.	Precision = 0.99

**Table 8:** The comparison of several typical machine learning-based surface detection approaches.

Taxonomy	Reference	Approaches	Strengths and Weaknesses
Supervised learning	[109]	A double-layer feed-forward neural network	Quite simple, effective and robust but dependent on labeled samples, and the number is limited
	[110]	Convolutional neural network (CNN) and Naive Bayesian data fusion schemes (NB-CNN)	
	[111]	Improved Fast R-CNN	
	[112]	Classification priority network (CPN)	
Unsupervised learning	[113]	Clustering	Requires no labeled samples for training but is susceptible to noise and highly influenced by initial values
	[37]	Convolutional automatic encoder	
Semi-supervised learning	[114]	Generative adversarial network (GAN)	Requires only a small number of labeled samples and the result is stable, but requires many interactions and reduces efficiency
	[8]	Convolutional auto-encoder (CAE) and semi-supervised GAN fusion schemes	
	[115]	Convolutional neural network based on a residual structure	

**Table 9:** The comparison of three-dimensional detection approaches.

Approaches	Reference	Advantage	Disadvantage
Stereoscopic vision	[140,141,142,143,144]	Suitable for areas with large texture variations and is very sensitive to normal surface disturbances	Depends on the intrinsic texture information of the object surface
Photometric stereo	[145,146]	There is no need to know the precise 3D relationship between the test object and the camera, or to use two cameras to capture 3D data	Limitations of detecting non-Lambertian surfaces such as glossy metals
Laser scanner	[147,148,149,150,151,152,153]	Reproduce the surface shape so that it is non-contact, non-destructive, and has high precision	The equipment cost is high and the calculation amount is large
Structural light	[154,155,156,157,158,159]	High spatial resolution and accuracy	Complex calculation and difficult to calibrate accurately

## References

[1] Li N., Wang F., Song G. (2020). New entropy-based vibro-acoustic modulation method for metal fatigue crack detection: An exploratory study. Measurement.

[2] Li Q., Chen J., Zhao L. (2020). Research on an improved metal surface defect detection sensor based on a 3D RFID tag antenna. J. Sens..

[3] Lv X., Duan F., Jiang J., Fu X., Gan L. (2020). Deep metallic surface defect detection: The new benchmark and detection network. Sensors.

[4] Lv X., Duan F., Jiang J., Fu X., Gan L. (2020). Deep active learning for surface defect detection. Sensors.

[5] Ghorai S., Mukherjee A., Gangadaran M., Dutta P.K. (2013). Automatic defect detection on hot-rolled flat steel products. IEEE Trans. Instrum. Meas..

[6] Song K., Yan Y. (2013). A noise robust method based on completed local binary patterns for hot-rolled steel strip surface defects. Appl. Surf. Sci..

[7] Luo Q., Sun Y., Li P., Simpson O., Tian L., He Y. (2019). Generalized completed local binary patterns for time-efficient steel surface defect classification. IEEE Trans. Instrum. Meas..

[8] He D., Xu K., Zhou P., Zhou D. (2019). Surface defect classification of steels with a new semi-supervised learning method. Opt. Lasers Eng..

[9] Luo Q., He Y. (2016). A cost-effective and automatic surface defect inspection system for hot-rolled flat steel. Robot. Comput. Integr. Manuf..

[10] Usamentiaga R., Garcia D.F., Molleda J., Bulnes F.G., Bonet Brana G. (2014). Vibrations in steel strips: Effects on flatness measurement and filtering. IEEE Trans. Ind. Appl..

[11] Inc R. RAMON Surface Quality Detection System for Continuous. http://www.ramon.com.cn.

[12] Zhou X., Wang Y., Zhu Q., Mao J., Xiao C., Lu X., Zhang H. (2020). A Surface defect detection framework for glass bottle bottom using visual attention model and wavelet transform. IEEE Trans. Ind. Inform..

[13] Jian C., Gao J., Ao Y. (2017). Automatic surface defect detection for mobile phone screen glass based on machine vision. Appl. Soft Comput..

[14] Chang F., Liu M., Dong M., Duan Y. (2019). A mobile vision inspection system for tiny defect detection on smooth car-body surfaces based on deep ensemble learning. Meas. Sci. Technol..

[15] Tsai D., Luo J. (2011). Mean shift-based defect detection in multicrystalline solar wafer surfaces. IEEE Trans. Ind. Inform..

[16] Zhang H., Qi X., Li X. Research on key technology of cold-rolled aluminum plate surface defect detection system. Proceedings of the 2nd International Conference on Mechatronics and Control Engineering (ICMCE 2013).

[17] Xuewu Z., Fang G., Lizhong X. (2012). Inspection of surface defects in copper strip using multivariate statistical approach and SVM. Int. J. Comput. Appl. Technol..

[18] Piironen T., Silven O., Pietikainen M., Laitinen T., Strommer E. (1990). Automated visual inspection of rolled metal surfaces. Mach. Vis. Appl..

[19] Keesug C., Kyungmo K., Lee J.S. Development of defect classification algorithm for POSCO rolling strip surface inspection system. Proceedings of the SICE-ICASE International Joint Conference.

[20] Rinn R., Becker M., Foehr R., Luecking F. Steel mill defect detection and classification at 3000 ft/min using mainstream technology. Proceedings of the Conference on Real-Time Imaging III.

[21] Zhang X., Ding Y., Lv Y., Shi A., Liang R. (2011). A vision inspection system for the surface defects of strongly reflected metal based on multi-class SVM. Expert Syst. Appl..

[22] Chin R.T., Harlow C.A. (1982). Automated visual inspection: A survey. IEEE Trans. Pattern Anal. Mach. Intell..

[23] Chin R.T. (1988). Automated visual inspection: 1981 to 1987. Comput. Vis. Graph. Image Process..

[24] Thomas A.D.H., Rodd M.G., Holt J.D., Neill C.J. (1995). Real-time industrial inspection: A review. Real-Time Imaging.

[25] Newman T.S., Jain A.K. (1995). A survey of automated visual inspection. Comput. Vis. Image Underst..

[26] Kumar A. (2008). Computer-vision-based fabric defect detection: A survey. IEEE Trans. Ind. Electron..

[27] Ngan H.Y.T., Pang G.K.H., Yung N.H.C. (2011). Automated fabric defect detection—A review. Image Vis. Comput..

[28] Huang S., Pan Y. (2015). Automated visual inspection in the semiconductor industry: A survey. Comput. Ind..

[29] Xie X. (2008). A review of recent advances in surface defect detection using texture analysis techniques. Electron. Lett. Comput. Vis. Image Anal..

[30] Luo J., Dong T., Song D., Xiu C. (2014). A review on surface defect detection. J. Front. Comput. Sci. Technol..

[31] Tang B., Kong J.Y., Wu S.Q. (2017). Review of surface defect detection based on machine vision. J. Image Graph..

[32] Czimmermann T., Ciuti G., Milazzo M., Chiurazzi M., Roccella S., Oddo C.M., Dario P. (2020). Visual-based defect detection and classification approaches for industrial applications-a survey. Sensors.

[33] Neogi N., Mohanta D.K., Dutta P.K. (2014). Review of vision-based steel surface inspection systems. Eurasip J. Image Video Process..

[34] Sun X., Gu J., Tang S., Li J. (2018). Research progress of visual inspection technology of steel products—A review. Appl. Sci..

[35] Luo Q., Fang X., Liu L., Yang C., Sun Y. (2020). Automated visual defect detection for flat steel surface: A survey. IEEE Trans. Instrum. Meas..

[36] Wang H., Zhang J., Tian Y., Chen H., Sun H., Liu K. (2019). A simple guidance template-based defect detection method for strip steel surfaces. IEEE Trans. Ind. Inform..

[37] Youkachen S., Ruchanurucks M., Phatrapornnant T., Kaneko H. Defect segmentation of hot-rolled steel strip surface by using convolutional auto-encoder and conventional image processing. Proceedings of the 10th International Conference of Information and Communication Technology for Embedded Systems (IC-ICTES).

[38] Wu H., Xu X., Gao W. Uneven illumination surface defects inspection based on convolutional neural network. Proceedings of the International Conference of Computer Vision and Pattern Recognition.

[39] Shi T., Kong J., Wang X., Liu Z., Zheng G. (2016). Improved sobel algorithm for defect detection of rail surfaces with enhanced efficiency and accuracy. J. Cent. South. Univ..

[40] Sharifzadeh M., Alirezaee S., Amirfattahi R., Sadri S. Detection of steel defect using the image processing algorithms. Proceedings of the Inmic: International Multitopic Conference, 12th IEEE International Multitopic Conference.

[41] Ma Y., Li Q., Zhou Y., He F., Xi S. (2017). A surface defects inspection method based on multidirectional gray-level fluctuation. Int. J. Adv. Robot. Syst..

[42] Wang Y., Xia H., Yuan X., Li L., Sun B. (2018). Distributed defect recognition on steel surfaces using an improved random forest algorithm with optimal multi-feature-set fusion. Multimed. Tools Appl..

[43] Huang X., Luo X. A real-time algorithm for aluminum surface defect extraction on non-uniform image from CCD camera. Proceedings of the International Conference on Machine Learning and Cybernetics (ICMLC).

[44] Borselli A., Colla V., Vannucci M., Veroli M. A fuzzy inference system applied to defect detection in flat steel production. Proceedings of the IEEE International Conference on Fuzzy Systems.

[45] Shen Y. (2010). Techniques of machine vision applied in detection of copper strip surface’s defects. Electron. Meas. Technol..

[46] Zhao J., Yan Y., Liu W., Tong J. (2010). A multi-scale edge detection method of steel strip surface defects online detection system. J. Northeast. Univ. Nat. Sci..

[47] Kulkarni R., Banoth E., Pal P. (2019). Automated surface feature detection using fringe projection: An autoregressive modeling-based approach. Opt. Lasers Eng..

[48] Bulnes F.G., Garcia D.F., Javier De la Calle F., Usamentiaga R., Molleda J. (2016). A non-invasive technique for online defect detection on steel strip surfaces. J. Nondestruct. Eval..

[49] Choi J., Kim C. Unsupervised detection of surface defects: A two-step approach. Proceedings of the 19th IEEE International Conference on Image Processing (ICIP).

[50] Djukic D., Spuzic S. (2007). Statistical discriminator of surface defects on hot rolled steel. Image Vis. Comput..

[51] Zhai M., Jing Z., Fu S., Luo X. (2009). Defect detection in aluminum foil by input-estimate-based chi-square detector. Opt. Eng..

[52] Timm F., Barth E. Non-parametric texture defect detection using Weibull features. Proceedings of the Conference on Image Processing—Machine Vision Applications IV.

[53] Liu K., Wang H., Chen H., Qu E., Tian Y., Sun H. (2017). Steel surface defect detection using a new haar-weibull-variance model in unsupervised manner. IEEE Trans. Instrum. Meas..

[54] Ojala T., Pietikainen M., Harwood D. (1996). A comparative study of texture measures with classification based on feature distributions. Pattern Recognit..

[55] Song K.C., Yan Y.H., Chen W.H., Zhang X. (2013). Research and perspective on local binary pattern. Acta Automatica Sinica.

[56] Wang J., Li Q., Gan J., Yu H., Yang X. (2020). Surface defect detection via entity sparsity pursuit with intrinsic priors. IEEE Trans. Ind. Inform..

[57] Chu M., Gong R. (2015). Invariant feature extraction method based on smoothed local binary pattern for strip steel surface defect. ISIJ Int..

[58] Liao S., Zhu X., Lei Z., Zhang L., Li S.Z. Learning multi-scale block local binary patterns for face recognition. Proceedings of the International Conference on Biometrics.

[59] Cao B., Li J., Qiao N. (2020). Nickel foam surface defect detection based on spatial-frequency multi-scale MB-LBP. Soft Comput..

[60] Liu Y., Xu K., Xu J. (2019). An improved MB-LBP defect recognition approach for the surface of steel plates. Appl. Sci..

[61] Luo Q., Fang X., Sun Y., Li L., Ai J., Yang C., Simpson O. (2019). Surface defect classification for hot-rolled steel strips by selectively dominant local binary patterns. IEEE Access.

[62] Haralick R.M., Shanmugam K., Dinstein I. (1973). Textural features for image classification. IEEE Trans. Syst. Man Cybern..

[63] Tsai D.-M., Chen M.-C., Li W.-C., Chiu W.-Y. (2012). A fast regularity measure for surface defect detection. Mach. Vis. Appl..

[64] Ai Y., Xu K. (2013). Surface detection of continuous casting slabs based on curvelet transform and kernel locality preserving projections. J. Iron Steel Res. Int..

[65] Choi D.C., Jeon Y.J., Yun J.P., Kim S.W. (2011). Pinhole detection in steel slab images using Gabor filter and morphological features. Appl. Opt..

[66] Wu X., Xu K., Xu J. Application of undecimated wavelet transform to surface defect detection of hot rolled steel plates. Proceedings of the 1st International Congress on Image and Signal Processing.

[67] Yazdchi M., Yazdi M., Mahyari A.G. Steel surface defect detection using texture segmentation based on multifractal dimension. Proceedings of the International Conference on Digital Image Processing (ICDIP).

[68] Paulraj M.P., Shukry A.M.M., Yaacob S., Adom A.H., Krishnan R.P. Structural steel plate damage detection using DFT spectral energy and artificial neural network. Proceedings of the 6th International Colloquium on Signal Processing & its Applications.

[69] Yun J.P., Choi S., Kim J.-W., Kim S.W. (2009). Automatic detection of cracks in raw steel block using Gabor filter optimized by univariate dynamic encoding algorithm for searches (uDEAS). NDT E Int..

[70] Park C., Bae H., Yun J., Yun S. The automated surface inspection system for hot slab. Proceedings of the 13th International Conference on Control, Automation and Systems (ICCAS).

[71] Wu H. (2016). Research of copper bar surface defects inspection system based on machine vision. Instrum. Tech. Sens..

[72] Chol D.C., Jeon Y.J., Kim S.H., Moon S., Yun J.P., Kim S.W. (2017). Detection of pinholes in steel slabs using gabor filter combination and morphological features. ISIJ Int..

[73] Medina R., Gayubo F., Gonzalez-Rodrigo L.M., Olmedo D., Gomez-Garcia-Bermejo J., Zalama E., Peran J.R. (2011). Automated visual classification of frequent defects in flat steel coils. Int. J. Adv. Manuf. Technol..

[74] Tolba A.S. (2011). Fast defect detection in homogeneous flat surface products. Expert Syst. Appl..

[75] Wu H., Xu X., Chu J., Duan L., Siebert P. (2019). Particle swarm optimization-based optimal real Gabor filter for surface inspection. Assem. Autom..

[76] Tolba A.S., Raafat H.M. (2015). Multiscale image quality measures for defect detection in thin films. Int. J. Adv. Manuf. Technol..

[77] Li X., Tso S.K., Guan X., Huang Q. (2006). Improving automatic detection of defects in castings by applying wavelet technique. IEEE Trans. Ind. Electron..

[78] Tsai D., Lin M. (2013). Machine-vision-based identification for wafer tracking in solar cell manufacturing. Robot. Comput. Integr. Manuf..

[79] Jeon Y., Yun J., Choi D., Kim S.W. Defect detection algorithm for corner cracks in steel billet using discrete wavelet transform. Proceedings of the ICROS-SICE International Joint Conference.

[80] Weiwei L., Yunhui Y. (2014). Automated surface defect detection for cold-rolled steel strip based on wavelet anisotropic diffusion method. Int. J. Ind. Syst. Eng..

[81] Song K., Hu S., Yan Y. (2014). Automatic recognition of surface defects on hot-rolled steel strip using scattering convolution network. J. Comput. Inf. Syst..

[82] Xu K., Song M., Yang C., Zhou P. (2013). Application of hidden markov tree model to on-line detection of surface defects for steel strips. J. Mech. Eng..

[83] Zhou S., Wu S., Liu H., Lu Y., Hu N. (2018). Double low-rank and sparse decomposition for surface defect segmentation of steel sheet. Appl. Sci..

[84] Cross G.R., Jain A.K. (1983). Markov random field texture models. IEEE Trans. Pattern Anal. Mach. Intell..

[85] Gayubo F., Gonzalez J.L., de la Fuente E., Miguel F., Peran J.R. On-line machine vision system for detect split defects in sheet-metal forming processes. Proceedings of the 18th International Conference on Pattern Recognition (ICPR 2006).

[86] Zhang X., Ding Y., Duan D., Fang G., Xu L., Shi A. (2011). Surface defects inspection of copper strips based on vision bionics. J. Image Graph..

[87] Zhiznyakov A.L., Privezentsev D.G., Zakharov A.A. (2015). Using fractal features of digital images for the detection of surface defects. Pattern Recognit. Image Anal..

[88] Shi B., Qiao P. (2018). A new surface fractal dimension for displacement mode shape-based damage identification of plate-type structures. Mech. Syst. Signal. Process..

[89] Zhang X., Li W., Xi J., Zhang Z., Fan X. (2013). Surface defect target identification on copper strip based on adaptive genetic algorithm and feature saliency. Math. Probl. Eng..

[90] Harel J., Koch C., Perona P. (2006). Graph-Based Visual Saliency.

[91] Tu W., He S., Yang Q., Chen S. Real-time salient object detection with a minimum spanning tree. Proceedings of the IEEE Conference on Computer Vision and Pattern Recognition (CVPR).

[92] Wang Q., Zheng W., Piramuthu R. GraB: Visual saliency via novel graph model and background priors. Proceedings of the 2016 IEEE Conference on Computer Vision and Pattern Recognition (CVPR).

[93] Yu H., Li Q., Tan Y., Gan J., Wang J., Geng Y.-A., Jia L. (2019). A coarse-to-fine model for rail surface defect detection. IEEE Trans. Instrum. Meas..

[94] Song Q., Oskoui E.A., Taylor T., Ansari F. (2019). Visual saliency-based image binarization approach for detection of surface microcracks by distributed optical fiber sensors. Struct. Health Monit. Int. J..

[95] Yan Y., Kaneko S.I., Asano H. (2020). Accumulated and aggregated shifting of intensity for defect detection on micro 3D textured surfaces. Pattern Recognit..

[96] Song G., Song K., Yan Y. (2020). EDRNet: Encoder-Decoder Residual network for salient object detection of strip steel surface defects. IEEE Trans. Instrum. Meas..

[97] Song G., Song K., Yan Y. (2020). Saliency detection for strip steel surface defects using multiple constraints and improved texture features. Opt. Lasers Eng..

[98] Zhang D., Song K., Xu J., He Y., Yan Y. (2020). Unified detection method of aluminium profile surface defects: Common and rare defect categories. Opt. Lasers Eng..

[99] Dong H., Song K., He Y., Xu J., Yan Y., Meng Q. (2019). PGA-Net: Pyramid feature fusion and global context attention network for automated surface defect detection. IEEE Trans. Ind. Inform..

[100] Celik T., Tjahjadi T. (2012). Automatic image equalization and contrast enhancement using gaussian mixture modeling. IEEE Trans. Image Process..

[101] Liu K., Luo N., Li A., Tian Y., Sajid H., Chen H. (2020). A new self-reference image decomposition algorithm for strip steel surface defect detection. IEEE Trans. Instrum. Meas..

[102] Zhou F., Liu G., Xu F., Deng H. (2019). A generic automated surface defect detection based on a bilinear model. Appl. Sci..

[103] Yu J., Lu X., Zong W.Z. (2016). Wafer defect detection and recognition based on local and nonlocal linear discriminant analysis and dynamic ensemble of gaussian mixture models. Acta Autom. Sin..

[104] Susan S., Sharma M. (2017). Automatic texture defect detection using Gaussian mixture entropy modeling. Neurocomputing.

[105] Yan H., Paynabar K., Shi J. (2017). Anomaly detection in images with smooth background via smooth-sparse decomposition. Technometrics.

[106] Huangpeng Q., Zhang H., Zeng X., Huang W. (2018). Automatic visual defect detection using texture prior and low-rank representation. IEEE Access.

[107] Duan Y.J., Lv Y.S., Zhang J., Zhao X.L., Wang Y.F. (2016). Deep learning for control: The state of the art and prospects. Acta Autom. Sin..

[108] Zhu Y., Zhao J.K., Wang Y.N., Zheng B.B. (2016). A review of human action recognition based on deep learning. Acta Autom. Sin..

[109] Kang G.W., Liu H.B. Surface defects inspection of cold rolled strips based on neural network. Proceedings of the 4th International Conference Machine Learning Cybernetics.

[110] Chen F., Jahanshahi M.R. (2018). NB-CNN: Deep learning-based crack detection using convolutional neural network and naive bayes data fusion. IEEE Trans. Ind. Electron..

[111] Zhou Z., Lu Q., Wang Z., Huang H. (2019). Detection of micro-defects on irregular reflective surfaces based on improved faster R-CNN. Sensors.

[112] He D., Xu K., Zhou P. (2019). Defect detection of hot rolled steels with a new object detection framework called classification priority network. Comput. Ind. Eng..

[113] Bulnes F.G., Usamentiaga R., Garcia D.F., Molleda J. (2012). Vision-based sensor for early detection of periodical defects in web materials. Sensors.

[114] Lian J., Jia W., Zareapoor M., Zheng Y., Luo R., Jain D.K., Kumar N. (2020). Deep-learning-based small surface defect detection via an exaggerated local variation-based generative adversarial network. IEEE Trans. Ind. Inform..

[115] Zheng X., Wang H., Chen J., Kong Y., Zheng S. (2020). A generic semi-supervised deep learning-based approach for automated surface inspection. IEEE Access.

[116] Gai X., Ye P., Wang J., Wang B. Research on defect detection method for steel metal surface based on deep Learning. Proceedings of the 5th Information Technology and Mechatronics Engineering Conference.

[117] Katsamenis I., Protopapadakis E., Doulamis A., Doulamis N., Voulodimos A. Pixel-level corrosion detection on metal constructions by fusion of deep learning semantic and contour segmentation. Proceedings of the IEEE Conference on Computer Vision and Pattern Recognition (CVPR).

[118] Choi D., Jeon Y., Lee S., Yun J., Kim S. (2014). Algorithm for detecting seam cracks in steel plates using a Gabor filter combination method. Appl. Opt..

[119] Ashour M.W., Khalid F., Halin A.A., Abdullah L.N., Darwish S.H. (2019). Surface defects classification of hot-rolled steel strips using multi-directional shearlet features. Arab. J. Sci. Eng..

[120] Yun J.P., Shin W.C., Koo G., Kim M.S., Lee C., Lee S.J. (2020). Automated defect inspection system for metal surfaces based on deep learning and data augmentation. J. Manuf. Syst..

[121] Neuhauser F.M., Bachmann G., Hora P. (2020). Surface defect classification and detection on extruded aluminum profiles using convolutional neural networks. Int. J. Mater. Form..

[122] Zhao Y.J., Yan Y.H., Song K.C. (2017). Vision-based automatic detection of steel surface defects in the cold rolling process: Considering the influence of industrial liquids and surface textures. Int. J. Adv. Manuf. Technol..

[123] Tao X., Zhang D., Ma W., Liu X., Xu D. (2018). Automatic metallic surface defect detection and recognition with convolutional neural networks. Appl. Sci..

[124] Goodfellow I., Pouget-Abadie J., Mirza M., Xu B., Warde-Farley D., Ozair S., Courville A., Bengio Y. (2014). Generative adversarial nets. arXiv.

[125] Niu S., Li B., Wang X., Lin H. (2020). Defect image sample generation with GAN for improving defect recognition. IEEE Trans. Autom. Sci. Eng..

[126] Berthelot D., Carlini N., Goodfellow I., Oliver A., Papernot N., Raffel C. MixMatch: A holistic approach to semi-supervised learning. Proceedings of the 33rd Conference on Neural Information Processing Systems (NeurIPS).

[127] He Y., Song K., Meng Q., Yan Y. (2020). An end-to-end steel surface defect detection approach via fusing multiple hierarchical features. IEEE Trans. Instrum. Meas..

[128] Gan J., Li Q., Wang J., Yu H. (2017). A hierarchical extractor-based visual rail surface inspection system. IEEE Sens. J..

[129] Sun K., Shen Z., Shi Y., Xu Z., Yuan L., Ni X. Non-destructive detection of small blowholes in aluminum by using laser ultrasonics technique. Proceedings of the 17th International Conference on Photothermal and Photoacoustic Phenomena.

[130] Xiao R., Wang J., Xie X., Guo F., Li X. (2019). 3D center segregation reconstruction of steel continuous casting slab. Steel Res. Int..

[131] Ricci M., Ficola A., Fravolini M.L., Battaglini L., Palazzi A., Burrascano P., Valigi P., Appolloni L., Cervo S., Rocchi C. (2013). Magnetic imaging and machine vision NDT for the on-line inspection of stainless steel strips. Meas. Sci. Technol..

[132] Ege Y., Bicakci S., Gunes H., Citak H., Coramik M. (2019). An application of BRANN and MFL methods: Determining crack type and physical properties on M5 steel sheets. Measurement.

[133] He Y., Pan M., Luo F., Tian G. (2011). Pulsed eddy current imaging and frequency spectrum analysis for hidden defect nondestructive testing and evaluation. NDT E Int..

[134] Kishore M.B., Park D.G., Jeong J.R., Kim J.Y., Jacobs L.J., Lee D.H. (2015). Detection of deep subsurface cracks in thick stainless steel plate. J. Magn..

[135] Harzallah S., Chabaat M. (2017). 3D-FEM computation and experimental study of eddy currents for characterization of surface cracks. Int. J. Struct. Integr..

[136] Soni A.K., Rao B.P. (2018). Lock-in amplifier based eddy current instrument for detection of sub-surface defect in stainless steel plates. Sens. Imaging.

[137] Pernkopf F., O’Leary P. (2003). Image acquisition techniques for automatic visual inspection of metallic surfaces. NDT E Int..

[138] Koch C., Brilakis I. (2011). Pothole detection in asphalt pavement images. Adv. Eng. Inform..

[139] Wen X., Song K., Niu M., Dong Z., Yan Y. (2017). 3D inspection technology combining passive stereo matching and active structured light for steel plate surface sample. Int. J. Surf. Sci. Eng..

[140] Wen X., Song K., Huang L., Niu M., Yan Y. (2019). Complex surface ROI detection for steel plate fusing the gray image and 3D depth information. Optik.

[141] Tsai Y.H., Tsai D.M., Li W.C., Chiu W.Y., Lin M.C. (2011). Surface defect detection of 3D objects using robot vision. Ind. Robot. Int. J. Robot. Res. Appl..

[142] Torok M.M., Golparvar-Fard M., Kochersberger K.B. (2014). Image-based automated 3D crack detection for post-disaster building assessment. J. Comput. Civ. Eng..

[143] Von Enzberg S., Al-Hamadi A. (2016). A multiresolution approach to model-based 3-D surface quality inspection. IEEE Trans. Ind. Inform..

[144] Niu M., Song K., Huang L., Yan Y., Meng Q. (2020). Unsupervised saliency detection of rail surface defects using stereoscopic images. IEEE Trans. Ind. Inform..

[145] Ren M., Wang X., Xiao G., Chen M., Fu L. (2019). Fast defect inspection based on data-driven photometric stereo. IEEE Trans. Instrum. Meas..

[146] Kang D., Jang Y.J., Won S. (2013). Development of an inspection system for planar steel surface using multispectral photometric stereo. Opt. Eng..

[147] Erkal B.G., Hajjar J.F. (2017). Laser-based surface damage detection and quantification using predicted surface properties. Autom. Constr..

[148] Reyno T., Marsden C., Wowk D. (2018). Surface damage evaluation of honeycomb sandwich aircraft panels using 3D scanning technology. NDT E Int..

[149] Pernkopf F. (2004). Detection of surface defects on raw steel blocks using Bayesian network classifiers. Pattern Anal. Appl..

[150] Landstrom A., Thurley M.J. (2012). Morphology-based crack detection for steel slabs. IEEE J. Sel. Top. Signal Process..

[151] Zhao L.M., Ouyang Q., Chen D.F., Wen L.Y. (2011). Surface defects inspection method in hot slab continuous casting process. Ironmak. Steelmak..

[152] Martin D., Guinea D.M., Garcia-Alegre M.C., Villanueva E., Guinea D. (2010). Multi-modal defect detection of residual oxide scale on a cold stainless steel strip. Mach. Vis. Appl..

[153] Wells L.J., Shafae M.S., Camelio J.A. (2016). Automated surface defect detection using high-density data. J. Manuf. Sci. Eng. Trans. Asme.

[154] Zhang B., Huang W., Wang C., Gong L., Zhao C., Liu C., Huang D. (2015). Computer vision recognition of stem and calyx in apples using near-infrared linear-array structured light and 3D reconstruction. Biosyst. Eng..

[155] Chu H.H., Wang Z.Y. (2016). A vision-based system for post-welding quality measurement and defect detection. Int. J. Adv. Manuf. Technol..

[156] Xiong Z., Li Q., Mao Q., Zou Q. (2017). A 3D laser profiling system for rail surface defect detection. Sensors.

[157] Cao X., Xie W., Ahmed S.M., Li C.R. (2020). Defect detection method for rail surface based on line-structured light. Measurement.

[158] Song L.M., Wang D.N. (2006). A novel grating matching method for 3D reconstruction. NDT E Int..

[159] Zhang X.L., Ouyang Q., Peng S., Zhao L.M. (2014). Continuous casting slab surface crack depth measurement using sinusoidal phase grating method. Ironmak. Steelmak..

[160] Wen X., Song K., Niu M., Dong Z., Yan Y. (2017). A three-dimensional inspection system for high temperature steel product surface sample height using stereo vision and blue encoded patterns. Optik.

